# An Antibody-Recruiting Molecule Enhances Fcγ Receptor-Mediated Uptake and Killing of Mycobacterial Pathogens by Macrophages

**DOI:** 10.1021/acsinfecdis.5c00097

**Published:** 2025-05-01

**Authors:** Priscilla Dzigba, Grisha A. T. Dekhtyar, Mary Jane Hartman, Kai J. Winstead-Leroy, Mallary C. Greenlee-Wacker, Benjamin M. Swarts

**Affiliations:** †Department of Chemistry and Biochemistry and ‡Department of Biology, 5649 Central Michigan University, Mount Pleasant, Michigan 48859, United States; § Biochemistry, Cell, and Molecular Biology Graduate Programs, 5649 Central Michigan University, Mount Pleasant, Michigan 48859, United States; ∥Biological Sciences Department and ⊥Chemistry and Biochemistry Department, 7173 California Polytechnic State University, San Luis Obispo, California 93407, United States

**Keywords:** Mycobacteria, cell labeling, antibody-recruiting molecule (ARM), immunotherapy, macrophages, antibodies, Fcγ receptor, trehalose

## Abstract

Mycobacteria, which include the infectious agents for tuberculosis (TB) and nontuberculous mycobacteria (NTM) disease, pose a critical health challenge due to traits that allow them to evade host immune clearance and antibiotic action. Toward a novel immunotherapy approach for mycobacteria, we previously reported an antibody-recruiting molecule (ARM) strategy to specifically modify the surface glycans of mycobacteria with exogenous haptens, marking the bacteria for opsonization by endogenous antibodies and enhancing the antibody-mediated immune response. We showed that the ARM, a trehalose-dinitrophenyl conjugate (Tre-DNP), exploited a conserved metabolic pathway to metabolically label the surface of nonpathogenic Mycobacterium smegmatis with DNP, recruited anti-DNP antibodies to the bacterial surface, and enhanced phagocytosis of the bacteria by THP-1 cells. Here, we extend these findings by investigating the ability of the Tre-DNP ARM strategy to increase macrophage-mediated phagocytosis and killing of different pathogenic mycobacterial species and interrogating mechanisms associated with the outcome. We show that Tre-DNP successfully modified the surface of multiple pathogens, including Mycobacterium tuberculosis and the NTM species Mycobacterium abscessus and Mycobacterium avium, and that phagocytosis and killing of intracellular bacteria by differentiated THP-1 cells is significantly enhanced for all species. Furthermore, we find that enhanced uptake is dependent upon the Fcγ receptor (FcγR) and enhanced killing correlates with sustained production of reactive oxygen species (ROS) and increased phagosome-lysosome fusion. Overall, our data demonstrate that Tre-DNP efficiently promotes ingestion of mycobacteria by human macrophages via the FcγR and enhances host effector responses against the pathogen. Thus, ARMs are tools that can be exploited for the purposes of (i) conducting mechanistic studies on immune recognition and elimination of mycobacterial pathogens and (ii) developing immune-targeting strategies against mycobacterial pathogens.

## Graphical abstract

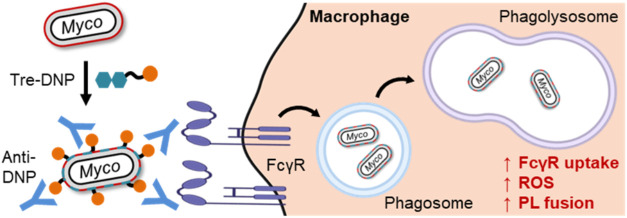

## Introduction

Mycobacteria are the primary cause of several pulmonary and extrapulmonary diseases that are refractory to treatment, most notably tuberculosis (TB) and nontuberculous mycobacteria (NTM) disease. According to the World Health Organization, Mycobacterium tuberculosis (Mtb) remains a critical public health concern due to its life-threatening impact, high global prevalence, and persistent treatment challenges.^1^ Every year, there are approximately 10 million new active TB infections and 1.3 million TB deaths.^1^ NTM, which comprise over 190 distinct species found in environmental settings, generally pose minimal risk to immunocompetent individuals.^2,3^ However, certain NTM species, such as Mycobacterium abscessus (Mabs) and Mycobacterium avium (M. avium), are opportunistic pathogens in chronic lung conditions like cystic fibrosis (CF), chronic obstructive pulmonary disease, and bronchiectasis, as well as immunocompromised individuals, including those with human immunodeficiency virus (HIV) resulting in severe clinical outcomes.^4^ The rate of incidence of NTM infections has risen steadily over the past decade.^2,5^ Due principally to the highly protective cell envelope and distinctive physiological characteristics of mycobacteria (e.g., biofilm formation, modification of target enzymes, efflux pumps, high mutation rates, dormancy),^6,7^ treatment of TB and NTM diseases requires extended antibiotic regimens lasting several months to years, often resulting in detrimental side effects and the emergence of drug resistance.^8–10^ These factors underscore the need for innovative therapeutic strategies to combat mycobacterial pathogens.

Immunotherapies are promising alternative treatments for treating mycobacterial infections, complementing traditional cellular immunity. Many species of mycobacteria facultative intracellular bacteria, capable of surviving extracellularly and replicating intracellularly. Although host susceptibility to disease depends on virulence, they share common features, such as the ability to survive and replicate within macrophages and evade immune defenses.^11–13^ For instance, in a protective response, macrophages kill bacteria by forming a phagosome around the ingested inoculum and synthesizing toxic reactive oxygen species (ROS) in this compartment or by trafficking the phagosome to the lysosome, where degradative enzymes and antimicrobial peptides eliminate the bacteria.^14^ However, mycobacteria have evolved several mechanisms to evade these responses, including antigenic variation that decreases ROS production and by manipulating signaling pathways in order to slow phagosome-lysosome fusion.^15^ For example, mannose-capped lipoarabinomannan (ManLAM) on the mycobacterial surface targets mycobacteria to mannose receptors on phagocytes and uptake through these receptors prevents phagolysosome fusion.^16,17^

Impaired killing by macrophages and failure to induce sterilizing immunity contribute to persistent infections, but immunotherapies have the potential to override these pathogenic mechanisms and stimulate protective host responses.^18,19^ Although cellular immunity and production of cytokines IL-12, interferon-γ (IFN-γ), and tumor necrosis factor-α (TNF-α) are central to controlling mycobacterial infections,^20^ emerging evidence highlights a protective role for antibodies.^21–24^ Prior work has demonstrated that antibodies increase phagocytosis, inhibit bacterial growth, and augment T-cell-mediated responses. For example, BCG-vaccine-induced IgG enhanced phagocytosis and reduced Mtb growth,^25^ and monoclonal antibodies targeting Mtb antigens (e.g., phosphate transporter subunit PstS1 and arabinomannan) promoted clearance of bacteria in an FcγR-dependent manner.^26^ Despite these promising findings, antibody-based approaches have not yet been fully developed as immunotherapeutic strategies against mycobacterial infections.

Given the potential of antibody-based therapies for addressing mycobacterial infections, we recently reported an immune-targeting strategy for mycobacteria centered on the use of antibody-recruiting molecules (ARMs).^27^ ARMs are bispecific small molecules consisting of (i) a target-binding terminus, which directs the ARM to specifically decorate the surface of a pathogenic target cell, and (ii) an antibody-binding terminus, which contains a hapten that recruits endogenous antibodies to the target cell, leading to opsonization and immune clearance.^28–33^ ARM strategies have been developed against several different target types, including cancer cells,^34–36^ viruses,^37^ fungi,^38^ and bacteria,^28–33,39–43^ including our recent work on mycobacteria.^27^ Our ARM strategy exploits unique trehalose glycolipids displayed on the cell surface of mycobacteria, which are not present in human cells or other types of bacteria and thus represent a mycobacteria-specific molecular target (Figure 1A).^44^ The initial ARM we developed is a trehalose-dinitrophenyl conjugate, Tre-DNP (Figure 1B), which exploits the mycobacteria-conserved trehalose glycolipid biosynthetic pathway to install DNP groups on the mycobacterial surface. Subsequently, anti-DNP antibodies, which exist endogenously in human serum,^45,46^ opsonize DNP-labeled mycobacteria and enhance macrophage effector functions (Figure 1C). Consistent with this design, our proof-of-concept study established that Tre-DNP selectively installs DNP groups onto the cell surface of the nonpathogenic model organism Mycobacterium smegmatis and enhances their phagocytosis by THP-1-derived macrophages in the presence of anti-DNP antibodies.^27^ However, several gaps remain with respect to establishing mechanistic understanding and translational relevance of mycobacteria-targeting ARMs. In the present study, we first demonstrate that, in addition to avirulent M. smegmatis, Tre-DNP efficiently labels the cell surface of multiple mycobacterial pathogens, including Mtb, Mabs, and M. avium, consistent with the trehalose glycolipid biosynthetic pathway being conserved across the *Mycobacterium* genus. Second, we show that the Tre-DNP ARM strategy enhances macrophage-mediated clearance of these pathogens, promoting not only significantly elevated phagocytosis, but also killing of phagocytosed mycobacteria. Third, our mechanistic studies reveal that these effects are driven by engagement of the FcγR, which triggers downstream increases in ROS production and phagosome-lysosome fusion. Taken together, this study extends our understanding of how bacteria-targeting ARMs operate and demonstrates the potential of Tre-DNP, and possibly other trehalose-based ARMs, as tools for investigating and manipulating the antibody-mediated immune response to mycobacterial infection.

**Figure 1:**
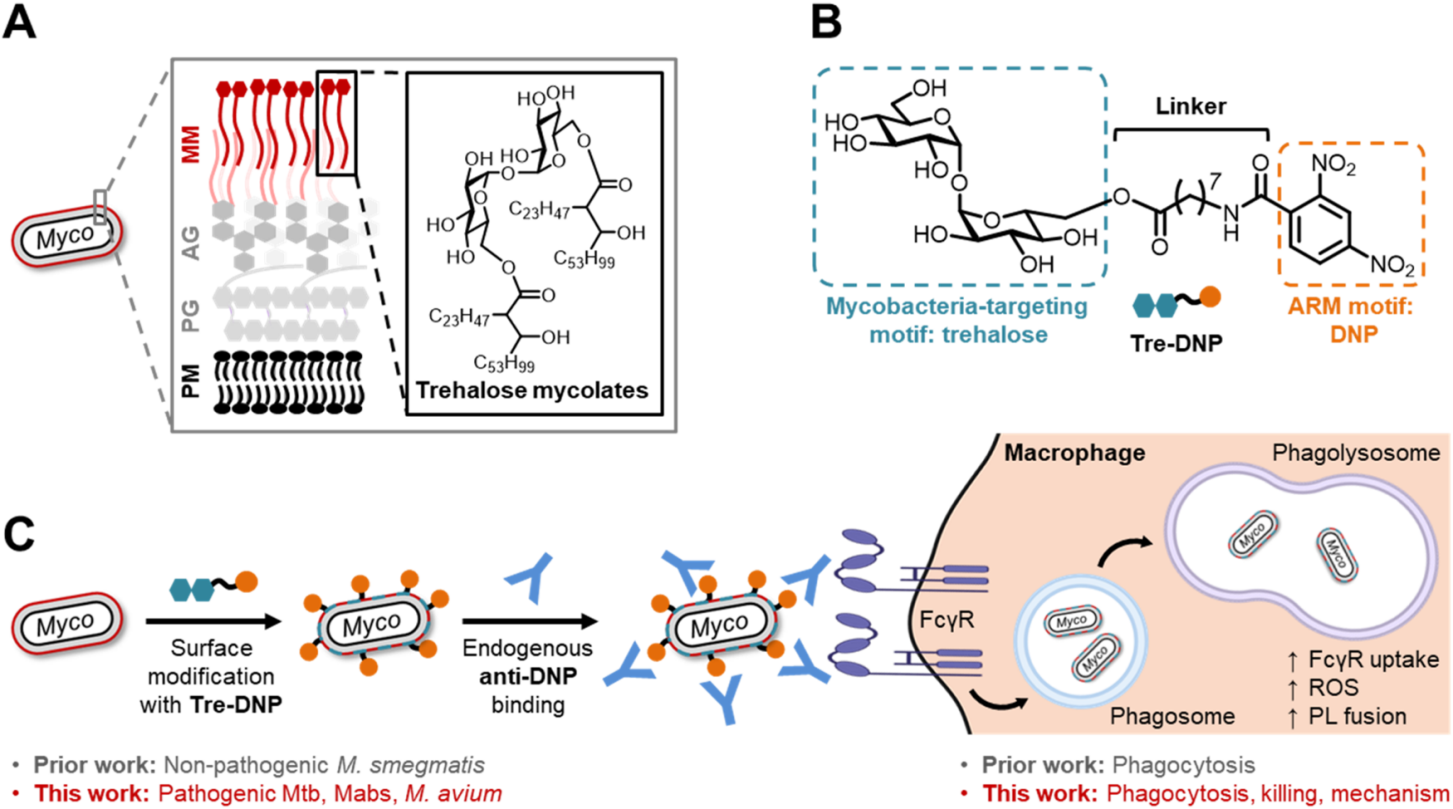
Antibody-recruiting molecule (ARM) strategy for mycobacteria. (A) The mycobacterial cell envelope contains surface-exposed trehalose glycolipids that are conserved in mycobacteria. (B) Structure of Tre-DNP, a mycobacteria-specific ARM consisting of a trehalose (Tre) targeting motif and a dinitrophenyl (DNP) antibody-recruiting motif. (C) Overview of ARM strategy, prior work, and focus of present study. Abbreviations: AG, arabinogalactan; MM, mycomembrane; PG, peptidoglycan; PM, plasma membrane. Figure adapted with permission from ref 27.

## Results

### Tre-DNP Labels and Recruits Anti-DNP Antibodies to Mycobacterial Pathogens

We previously showed that our first-generation ARM, Tre-DNP, metabolically incorporates into the cell surface of M. smegmatis through the action of antigen 85 (Ag85) mycoloyltransferases,^27^ which are extracellular enzymes that catalyze the transfer of mycolic acid lipids onto trehalose or trehalose derivatives to generate surface-exposed trehalose mycolates.^47^ Since trehalose mycolates and the Ag85 pathway are conserved in all mycobacteria, we reasoned that Tre-DNP would effectively incorporate into pathogenic mycobacterial species to enable opsonization by anti-DNP antibodies. To test this hypothesis, we evaluated Tre-DNP labeling of three mycobacterial pathogens with human health relevance, including Mtb, which causes TB, and Mabs and M. avium, which cause NTM disease. For the Mtb strain, we utilized mc^2^7000, a pantothenate auxotroph that can be handled safely in Biosafety Level 2 (BSL-2) containment.^48^ The different species were cultured in 7H9 liquid medium for approximately one doubling time in varying concentrations of Tre-DNP (or left untreated), then labeled with mouse anti-DNP IgG primary antibody or a control mouse isotype-matched IgG antibody and stained with antimouse IgG secondary antibody conjugated to phycoerythrin (PE) fluorophore. Flow cytometry analysis revealed that Tre-DNP efficiently installed DNP onto the surface of all three species in a dose-dependent manner, that the DNP groups were accessible to and bound by anti-DNP antibodies, and that anti-DNP antibody binding was specific for Tre-DNP-labeled bacteria (Figure 2A–C). Interestingly, the fold-change increase in fluorescence in Tre-DNP-treated versus untreated control was comparable in the three organisms, plateauing at 25–35-fold, implying a similar ability to integrate the compound into the cell surface. Several factors may have influenced the subtle variation observed in labeling efficiency across the different organisms. Differences in mycoloyltransferase substrate tolerance and/or enzymatic activity across species could impact the incorporation efficiency of Tre-DNP.^49^ Additionally, variations in cell envelope composition and organization may modulate the permeation of Tre-DNP and/or the accessibility of the DNP group on the bacterial surface, potentially influencing the extent of antibody binding and subsequent immune engagement.^50^ These results, combined with our published data showing that Tre-DNP does not label mammalian cells or representative Gram-positive or Gram-negative bacterial species,^27^ suggest that the Tre-DNP ARM strategy is suitable for specific targeting of mycobacteria, including pathogens, and that impact on host cells or microbiota should be limited.

**Figure 2:**
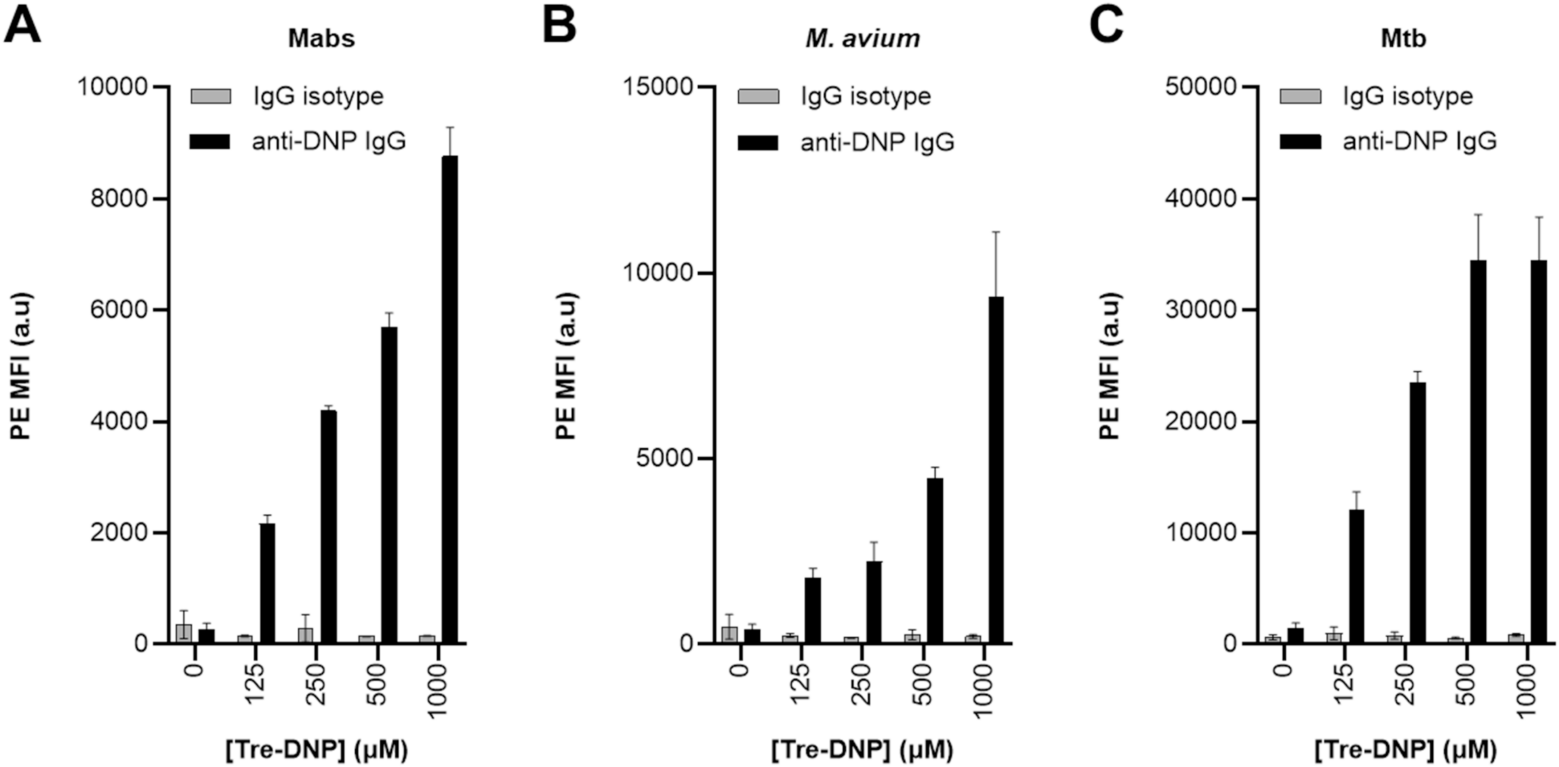
Tre-DNP incorporates into the cell surface of mycobacterial pathogens. *Mabs* (A), M. avium (B), or Mtb mc^2^7000 (C) were labeled with Tre-DNP at the specified concentrations (or left unlabeled) for 4, 12, and 24 h, respectively. Bacteria were washed, treated with mouse anti-DNP antibody (or mouse isotype control antibody), washed, and stained with PE-conjugated rabbit antimouse IgG antibody. Stained cells were fixed, washed, and analyzed by flow cytometry. Data are presented as means with error bars denoting standard deviation of three replicate experiments.

### Tre-DNP Enhances Antibody-Mediated, Fcγ Receptor-Dependent Phagocytosis of Mycobacterial Pathogens by Macrophages

Next, we sought to evaluate whether Tre-DNP-labeled, anti-DNP-opsonized mycobacterial pathogens undergo elevated phagocytosis by differentiated THP-1 cells that resemble macrophages. To quantitatively track phagocytosis of the different mycobacterial species without the need for genetic manipulation, we stained bacterial cells with 5-chloromethyl fluorescein diacetate (CMFDA), a cell-permeant fluorogenic dye that is taken up by cells and converted into a cell-impermeant fluorescent dye by cytoplasmic esterases in viable cells.^51^ First, we validated CMFDA staining in Mtb mc^2^7000, Mabs, and M. avium. We treated Tre-DNP-labeled bacteria with or without CMFDA, incubated with mouse anti-DNP antibodies, stained with PE-conjugated antimouse IgG antibodies, and analyzed by flow cytometry. Representative data for Mabs are shown in Figure 3 (see Figure S1 for flow cytometry dot plots), and data for M. avium and Mtb are shown in the Supporting Information (Figures S2 and S3). Virtually all of the CMFDA-treated bacteria were CMFDA^+^, while unstained bacteria exhibited minimal fluorescent signal in in the channel corresponding to CMFDA (Figures 3A, S2A, and S3A). In addition, CMFDA-treated and -untreated, Tre-DNP-labeled bacteria had a similar percentage of PE^+^ cells (Figures 3B, S2B, and S3B), confirming that anti-DNP antibody recruitment to the Tre-DNP surface label remained unaffected by CMFDA staining.

**Figure 3:**
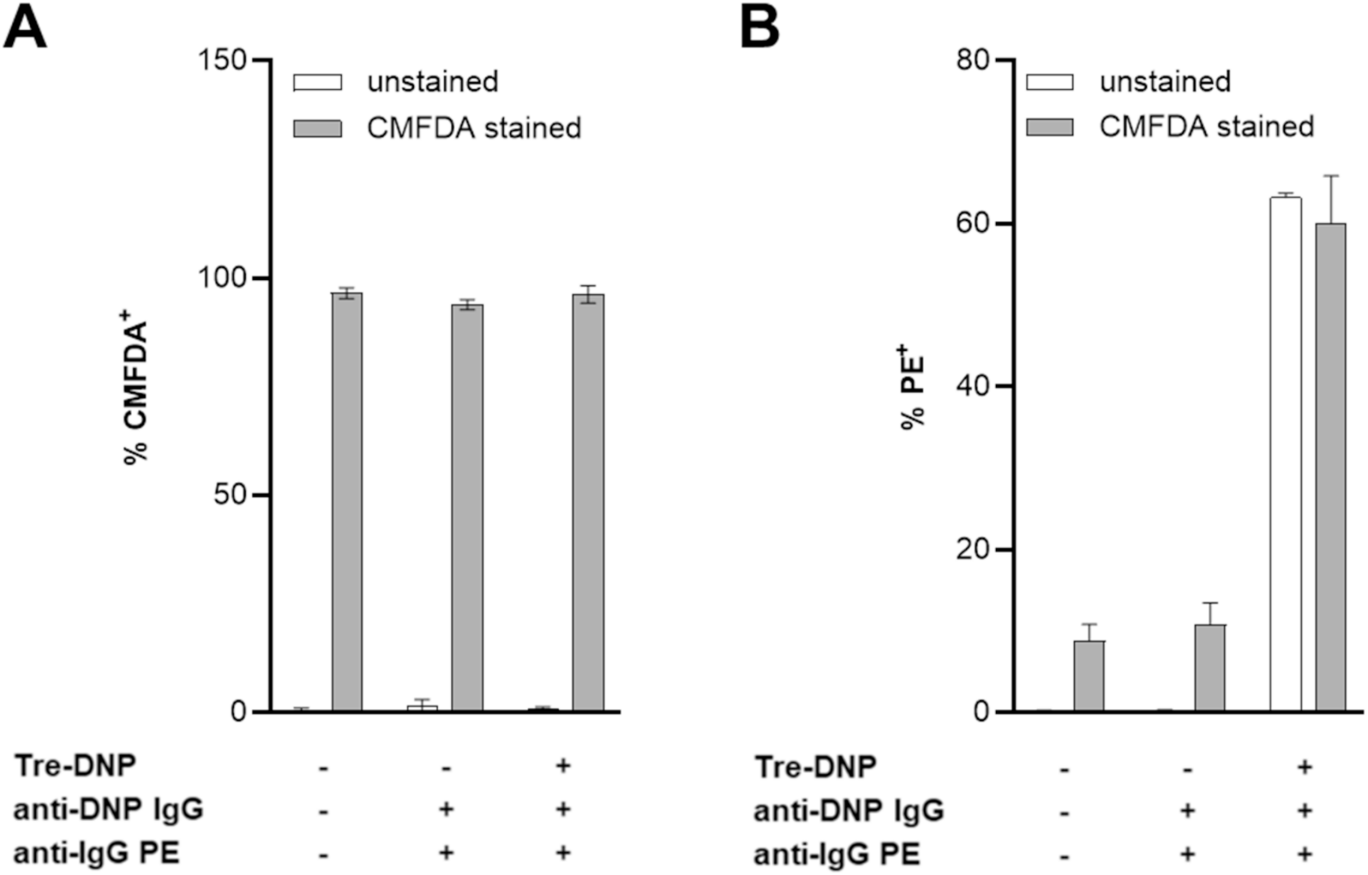
CMFDA stains mycobacterial pathogens without affecting Tre-DNP labeling. Mabs was labeled with Tre-DNP (250 μM) (or left unlabeled), then washed and stained with CMFDA (10 μM) (or left unstained). Bacteria were washed, treated with mouse anti-DNP antibody (or left untreated), washed, and stained with PE-conjugated rabbit antimouse IgG antibody. Cells were fixed, washed, and analyzed by flow cytometry. (A) Percentage of the population positive for fluorescein (CMFDA^+^), representing CMFDA-stained cells. (B) Percentage of the population positive for PE (PE^+^), representing antibody-bound cells. Data are presented as means with error bars denoting standard deviation of three replicate experiments.

Next, we tested whether Tre-DNP-labeled Mtb, Mabs, and M. avium opsonized with anti-DNP antibodies underwent elevated phagocytosis by differentiated THP-1 cells that resemble macrophages. Using an adapted method from our prior study,^27^ we cocultured phorbol 12-myristate 13-acetate (PMA)-differentiated THP-1 cells with CMFDA-stained, Tre-DNP-labeled, and anti-DNP antibody-opsonized mycobacteria at a multiplicity of infection (MOI) of 10, then harvested and fixed the cells. Differentiated THP-1 phenotypically resembled adherent macrophages and were identified by staining of cell surface marker CD14 with anti-CD14 antibody conjugated to allophycocyanin (APC).^52^ Flow cytometry was used to measure the percentage of differentiated THP-1 cells that engulfed CMFDA-stained bacteria (see Figure S4 for representative flow cytometry contour plots and gating strategy). Baseline uptake of unlabeled and nonopsonized Mabs, M. avium, and Mtb by THP-1 cells 12, 20, and 29%, respectively (Figure 4A–C). For Mabs and M. avium, uptake of Tre-DNP-labeled and anti-DNP-opsonized bacteria significantly increased by approximately 2-fold for both species compared to baseline (Figure 4A,4B); for Mtb, uptake significantly increased by approximately 1.7-fold (Figure 4C). THP-1 cells did not exhibit enhanced uptake of unlabeled or nonopsonized bacteria (Figure 4A–C). Together, our data demonstrate that Tre-DNP enhances phagocytosis by leveraging the opsonization capability of anti-DNP antibodies to promote antibody-dependent cellular phagocytosis (ADCP).

**Figure 4:**
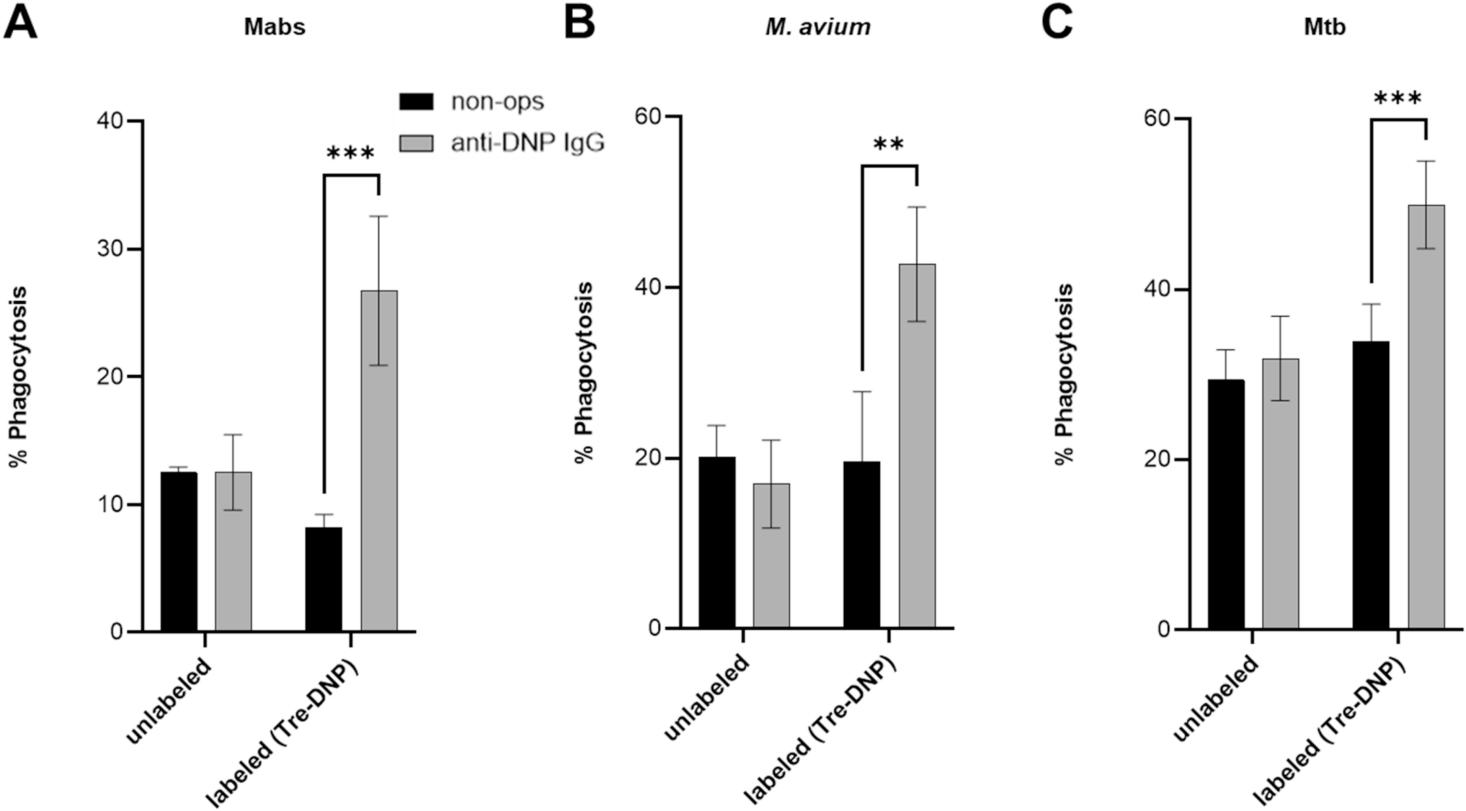
Tre-DNP enhances phagocytosis of mycobacterial pathogens by macrophages. Mabs, M. avium, or Mtb mc^2^7000 were labeled with Tre-DNP (250 μM) (or left unlabeled), washed, stained with CMFDA (10 μM), washed, and treated with mouse anti-DNP antibody (or left untreated). Bacteria (1 × 10^7^ CFU/mL) were cocultured with PMA-differentiated THP-1 cells (1 × 10^6^ cells/mL). After staining differentiated THP-1 cells with anti-CD14-APC, phagocytosis was analyzed by flow cytometry and calculated as CD14^+^CMFDA^+^ cells/total CD14^+^ cells × 100%. (A–C) Phagocytosis results for Mabs (A), M. avium (B), and Mtb mc^2^7000 (C). Error bars represent the standard deviation from three independent experiments. Statistical analysis was performed using a two-way ANOVA with Sidak’s multiple comparison test (*** *p* ≤ 0.001, ** *p* ≤ 0.01).

Macrophage cell-surface FcγRs are known to activate immune effector functions, including phagocytosis of antibody-opsonized bacteria by binding to the Fc region of IgG antibodies.^53,54^ For example, FcγR-dependent phagocytosis of Mtb was previously reported when bacteria were opsonized with IgG antibodies against the endogenous surface antigens PstS1 and arabinomannan.^26,55^ Therefore, we hypothesized that FcγRs mediate Tre-DNP-facilitated phagocytosis of mycobacterial pathogens. To test this hypothesis, we carried out phagocytosis experiments essentially as described above, but exposed THP-1 macrophages to a polyclonal FcγR- blocking antibody^56^ prior to infection with bacteria or left macrophages unexposed to FcγR blocker as a control. In the absence of FcγR blocker, Tre-DNP-labeled, anti-DNP-opsonized Mabs, M. avium, and Mtb mc^2^7000 underwent significantly increased phagocytosis by macrophages compared to nonopsonized conditions (Figure 5), consistent with the results shown above (Figure 4) (see Figure S5 for representative flow cytometry contour plots and gating strategy). However, pretreatment of macrophages with FcγR blocker significantly reduced phagocytosis of Tre-DNP-labeled, anti-DNP-opsonized bacteria to baseline levels for all species (Figure 5). These results indicate that the observed enhanced phagocytosis of Tre-DNP-labeled, anti-DNP-opsonized mycobacteria relies on interactions between FcγR and the opsonized bacteria, providing insight into the mechanism by which Tre-DNP promotes an immune effector response against pathogenic mycobacteria.

**Figure 5:**
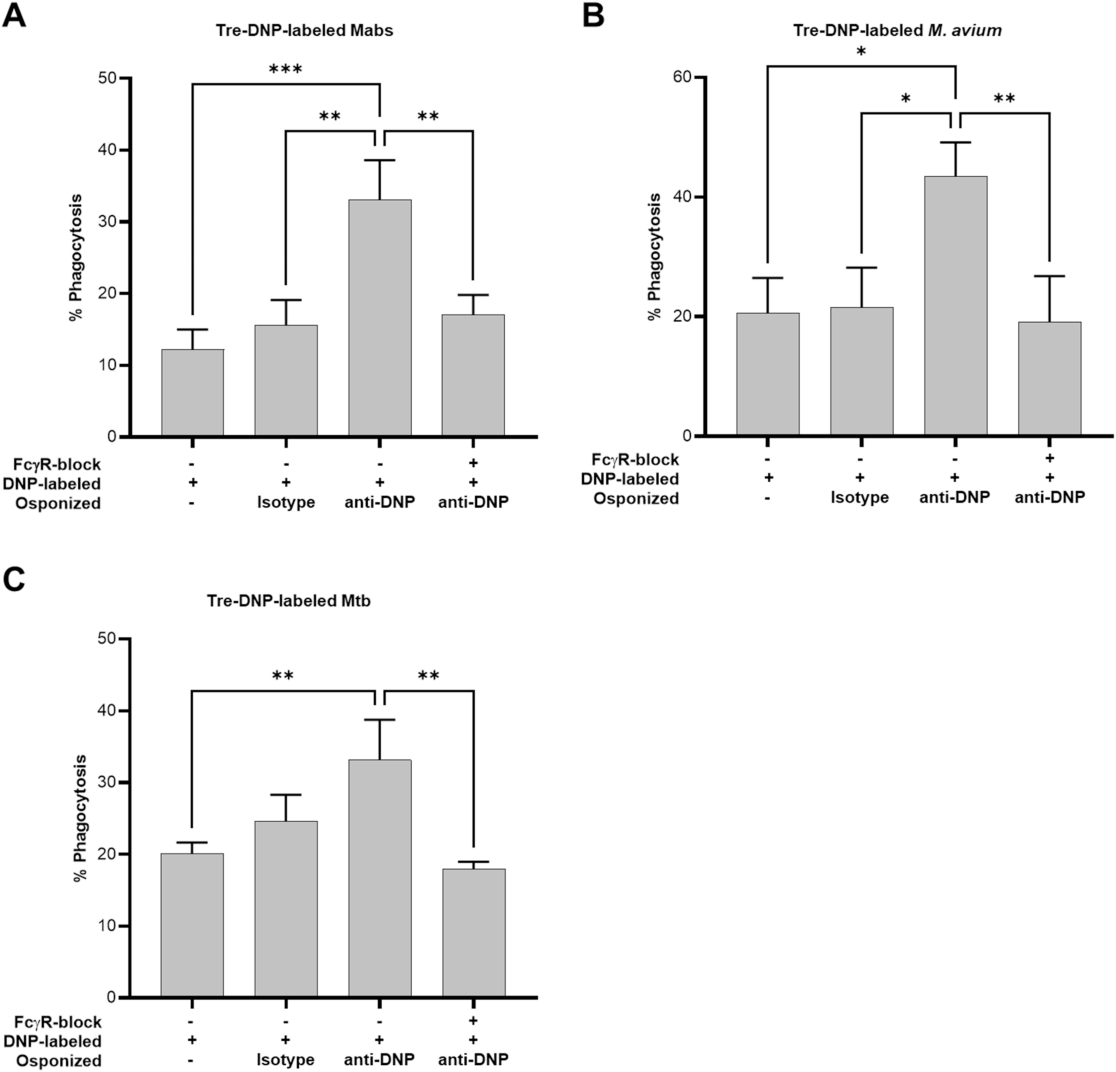
Enhancement of Tre-DNP-mediated phagocytosis of mycobacterial pathogens is FcγR-dependent. Mabs, M. avium, or Mtb mc^2^7000 were labeled with Tre-DNP (250 μM) (or left unlabeled), washed, stained with CMFDA (10 μM), washed, and left untreated or opsonized with antibody (either mouse anti-DNP IgG antibody or isotype IgG control antibody). Bacteria (1 × 10^7^ CFU/mL) were cocultured with PMA-differentiated THP-1 cells (1 × 10^6^ cells/mL). For FcγR blocking, PMA-differentiated THP-1 cells were pretreated with FcγR blocker for 30 min prior to coculturing with bacteria. After staining differentiated THP-1 cells with anti-CD14-APC, phagocytosis was analyzed by flow cytometry and calculated as CD14^+^CMFDA^+^ cells/total CD14^+^ cells × 100%. (A–C) Phagocytosis results for Mabs (A), M. avium (B), and Mtb mc^2^7000 (C). Error bars represent the standard deviation from three independent experiments. Statistical analysis was performed using a one-way ANOVA with Tukey’s multiple comparison test (*** *p* ≤ 0.001, ** *p* ≤ 0.01, * *p* ≤ 0.05).

### Tre-DNP Labeling and Opsonization Enhance the Killing of Mabs and M. avium by THP-1 Cells

Based on our observations of enhanced phagocytosis, we next investigated whether this increased uptake translated into increased intracellular killing of mycobacteria. To test this, PMA-differentiated THP-1 cells were cocultured with Mabs and M. avium and colony-forming units (CFU) were enumerated to assess bacterial survival. Mtb mc^2^7000 was excluded from these experiments due to its auxotrophic nature, which limits its suitability for growth-based assays. First, we accounted for increased uptake and a higher initial inoculum in THP-1 cells challenged with Tre-DNP-labeled and anti-DNP-opsonized bacteria (Figures 4 and 5) by calculating a replication index, a measure of killing or growth compared to the initial time point after phagocytosis.^57^ Overall, THP-1 cells more effectively eliminated Tre-DNP-labeled and anti-DNP-opsonized bacteria compared unlabeled or nonopsonized bacteria (Figure 6). For Mabs, a consistent decrease in the replication index of Tre-DNP-labeled, anti-DNP antibody-opsonized bacteria was observed at 48 h, while none of the other groups showed a similar reduction at this time point (Figure 6A). By 72 h, killing, indicated by a replication index lower than 1, was observed across all groups. However, the replication index for Tre-DNP-labeled, anti-DNP antibody-opsonized Mabs was significantly lower than that of Tre-DNP-labeled, nonopsonized bacteria, indicating enhanced bacterial clearance in the presence of both Tre-DNP labeling and antibody opsonization. Similarly, for M. avium, killing of Tre-DNP-labeled and anti-DNP antibody-opsonized bacteria occurred sooner than control groups and was significantly lower than Tre-DNP-labeled, nonopsonized bacteria after 48 h (Figure 6B). Again, by 72 h, bacterial killing was observed across all groups, which could be explained by reduced THP-1 cell viability and loss of an intracellular niche, entry of antibiotic into intracellular compartments,^58^ or activation of macrophage effector mechanisms. To exclude THP-1 cell death as a contributing factor to bacterial clearance, cell viability was assessed using the Alamar Blue assay. At 42 and 72 h, when bacterial killing was observed, there was no decrease in the viability of infected THP-1 cells (Figure S6), confirming that the reduction in CFU was independent of THP-1 cell death. These findings suggest that the opsonization of Tre-DNP-modified mycobacteria promotes the elimination of pathogenic mycobacteria within macrophages and aligns with research showing that antibodies against mycobacterial surface antigens can enhance bacterial killing in various models, including in vitro, ex vivo, and animal studies.^26,55,59,60^

**Figure 6:**
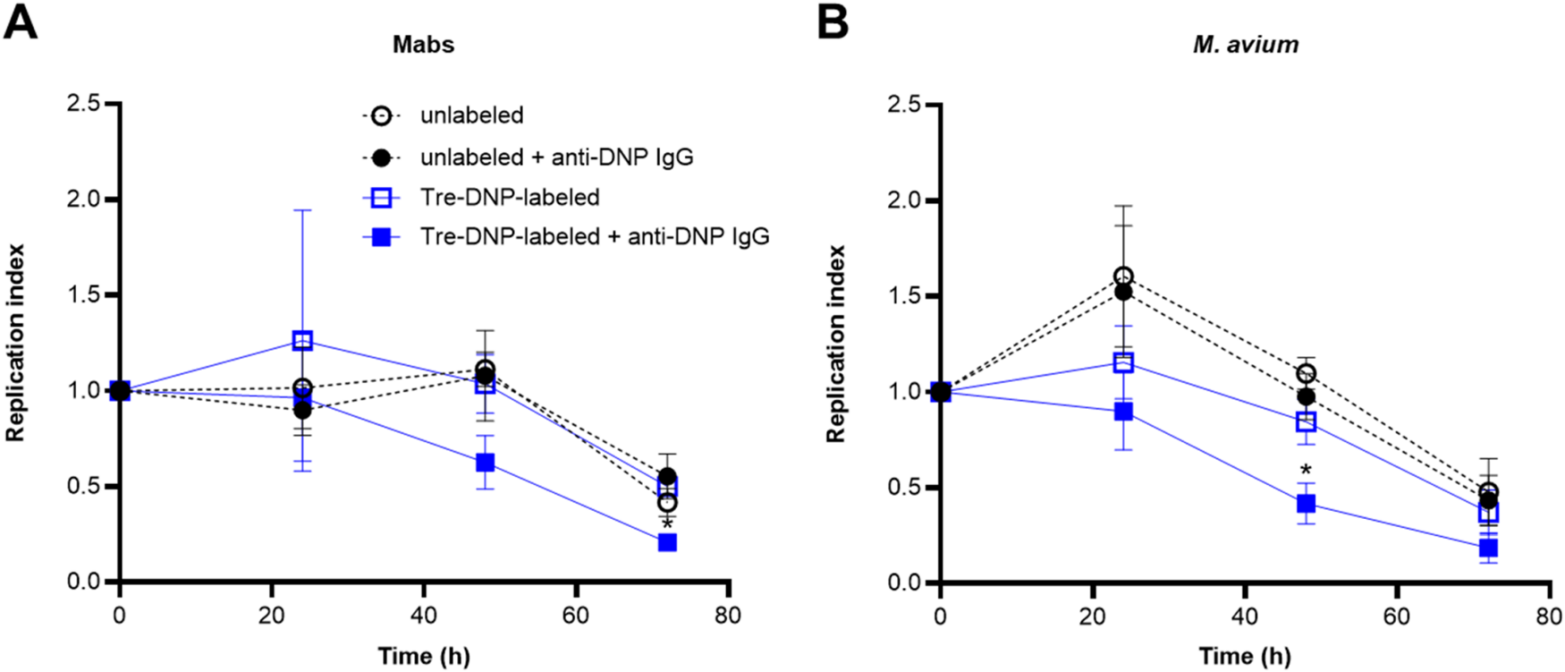
Tre-DNP enhances intracellular killing of Mabs and M. avium. Mabs (A) and M. avium (B) were either left unlabeled or Tre-DNP-labeled, and then treated with or without mouse anti-DNP antibodies (anti-DNP IgG). Bacteria (2 × 10^6^ CFU/mL) were cocultured with PMA-differentiated THP-1 cells (2 × 10^5^ cells/mL) for 1 h. Extracellular bacteria were eliminated with 200 μg/mL amikacin, and intracellular bacteria were recovered by lysing cells at the indicated time points. CFU was determined by plating serial dilations of lysates on 7H10 agar, and the replication index^57^ was calculated as CFU at a given time point/CFU at 0 h. Error bars represent the standard error of the mean from four independent experiments. Statistical analysis was performed on grouped data using a two-way ANOVA with Tukey’s multiple comparisons test (* *p* ≤ 0.05 vs unlabeled bacteria at respective time points).

### Mycobacteria Decorated with Tre-DNP and Antibody Trigger Sustained ROS Production by THP-1 Cells

ROS, which are primarily generated from the NADPH oxidase in macrophages, play an important role in the killing of engulfed pathogens.^61,62^ For instance, upon phagocytosis, the NADPH oxidase enzyme complex completes its assembly on the phagosome membrane, converts oxygen (O_2_) into superoxide (O_2_^•–^), and superoxide and its products directly damage bacterial membranes, proteins, and DNA.^61,63^ Given that Tre-DNP-labeled and anti-DNP-opsonized mycobacteria were killed more effectively by THP-1 cells, we investigated whether ROS production corresponded to increased killing. To assess ROS production, we used 2′,7′-dichlorodihydrofluorescein diacetate (H_2_DCFDA), a cell-permeable probe that is oxidized by some superoxide products to produce fluorescent 2′,7′-dichlorofluorescein (DCF). Irrespective of infection or treatment, DCF fluorescence, a measure of ROS production, decreased over time in differentiated THP-1 cells (Figure 7A,C). However, at later time points, ROS levels remained slightly higher in THP-1 cells challenged with Tre-DNP-labeled and anti-DNP opsonized bacteria. To evaluate this trend and the effect of blocking FcγRs, we normalized DCF values to uninfected THP-1 cells at each time point. Although no correlations between ROS production and increased killing were observed within the first 24 h, by 48 h, trends in ROS production differed between groups. For both Mabs and M. avium, ROS production was higher in THP-1 cells challenged with DNP-labeled and anti-DNP opsonized bacteria and it was significantly diminished when FcγRs were blocked (Figure 7B,D). These results indicate that enhanced killing is linked to sustained ROS production, and that blocking FcγRs on THP-1 cells interferes with this process. In our study, we did not observe a robust respiratory burst immediately after phagocytosis, and this could suggest that M. avium and Mabs entered differentiated THP-1 cells silently,^64^ or more likely, that differentiated THP-1 cells are still responding to PMA or another environmental factor with a maximum respiratory burst. However, the prolonged ROS production in THP-1 cells challenged with antibody-opsonized DNP-labeled mycobacteria corresponds to increased bacterial clearance, and given that oxidative killing mechanisms can contribute to NTM elimination,^65^ this work provides mechanistic insights into the efficacy of ARM.

**Figure 7:**
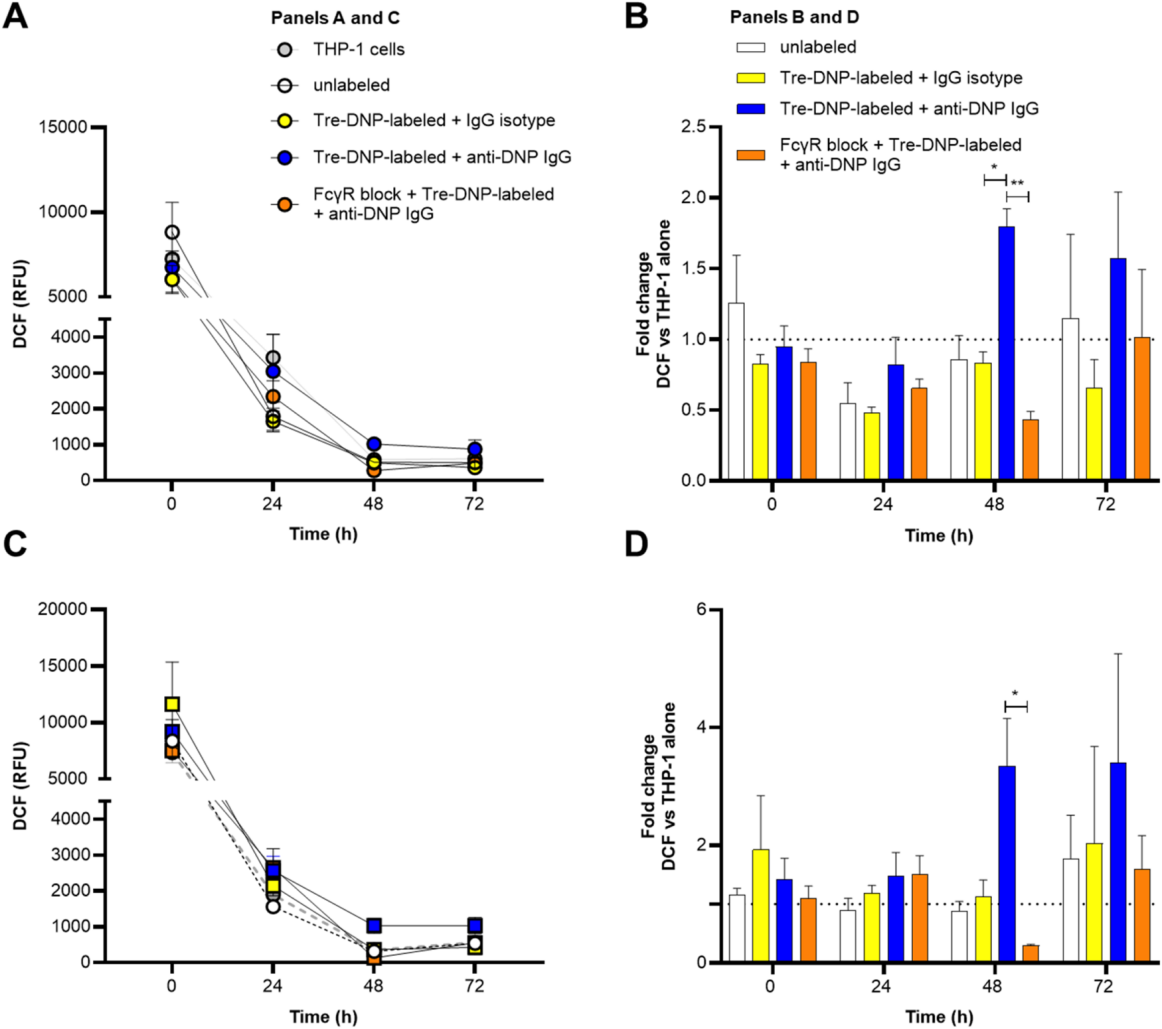
Tre-DNP-labeled and anti-DNP-opsonized Mabs and M. avium sustain ROS production in an FcγR-dependent manner. Mabs (A, B) and M. avium (C, D) were either labeled with Tre-DNP or left unlabeled. Bacteria were then incubated in buffered saline or opsonized with antibody (isotype or anti-DNP). The bacteria (2 × 10^6^ CFU/mL) were cocultured with PMA-differentiated THP-1 cells (2 × 10^5^ cells/mL) for 1 h, and extracellular bacteria were eliminated with 200 μg/mL amikacin. At each time point, ROS levels were detected using 10 μM H_2_DCFDA, and oxidation to DCF was measured in relative fluorescent units (RFU) (A, C). Fold-change in DCF was calculated as DCF RFU/DCF RFU of uninfected THP-1 cells at each time point (B, D). Error bars represent the standard error of the mean from three independent experiments. Statistical analysis was performed using a two-way ANOVA with Tukey’s multiple comparison test (** *p* ≤ 0.01, * *p* ≤ 0.05).

### Tre-DNP Anti-DNP Antibodies Promote Phagolysosome Fusion

Upon phagocytosis, bacteria are compartmentalized in a phagosome, which traffics to the lysosome for destruction. Delayed phagolysosome (PL) fusion is a hallmark of mycobacterial immune evasion, yet Fc-mediated uptake has been shown to increase PL fusion.^21,66,67^ On the basis of this rationale, we also investigated whether Tre-DNP labeling and anti-DNP antibody opsonization enhanced PL fusion in macrophages infected with Mabs. For these experiments, Mabs were chosen as a representative NTM for their clinical significance and more rapid growth, and phagolysosome fusion was quantified by assessing colocalization of CMFDA-labeled Mabs with LysoTracker, a pH-sensitive fluorescent dye that accumulates in acidic compartments, such as lysosomes and phagolysosomes. Given their stronger adherence and flatter morphology, J774 murine macrophages were used for imaging experiments instead of differentiated THP-1 cells. As demonstrated before, macrophages infected with Tre-DNP-labeled and anti-DNP-opsonized bacteria exhibited greater phagocytosis (Figures 8A and S7). To account for increased engulfment when Mabs was Tre-DNP-labeled and opsonized, the overlap of CMFDA with LysoTracker was quantified. After 1 h, there was more colocalization of Tre-DNP and opsonized Mabs with LysoTracker when compared to unlabeled and opsonized bacteria. Some comparisons did not reach statistical significance despite observed trends, possibly due to inherent variability in phagolysosome fusion dynamics.^68^ A similar pattern in PL fusion was observed after 4 h and there was a significant increase in colocalization between Mabs and LysoTracker when compared to macrophages infected with Tre-DNP-labeled bacteria that were not opsonized (Figure 8A,B). These findings suggest that both Tre-DNP labeling and anti-DNP opsonization synergistically promote phagolysosome fusion and underscore the potential of this approach for enhancing the immune response to mycobacteria.

**Figure 8:**
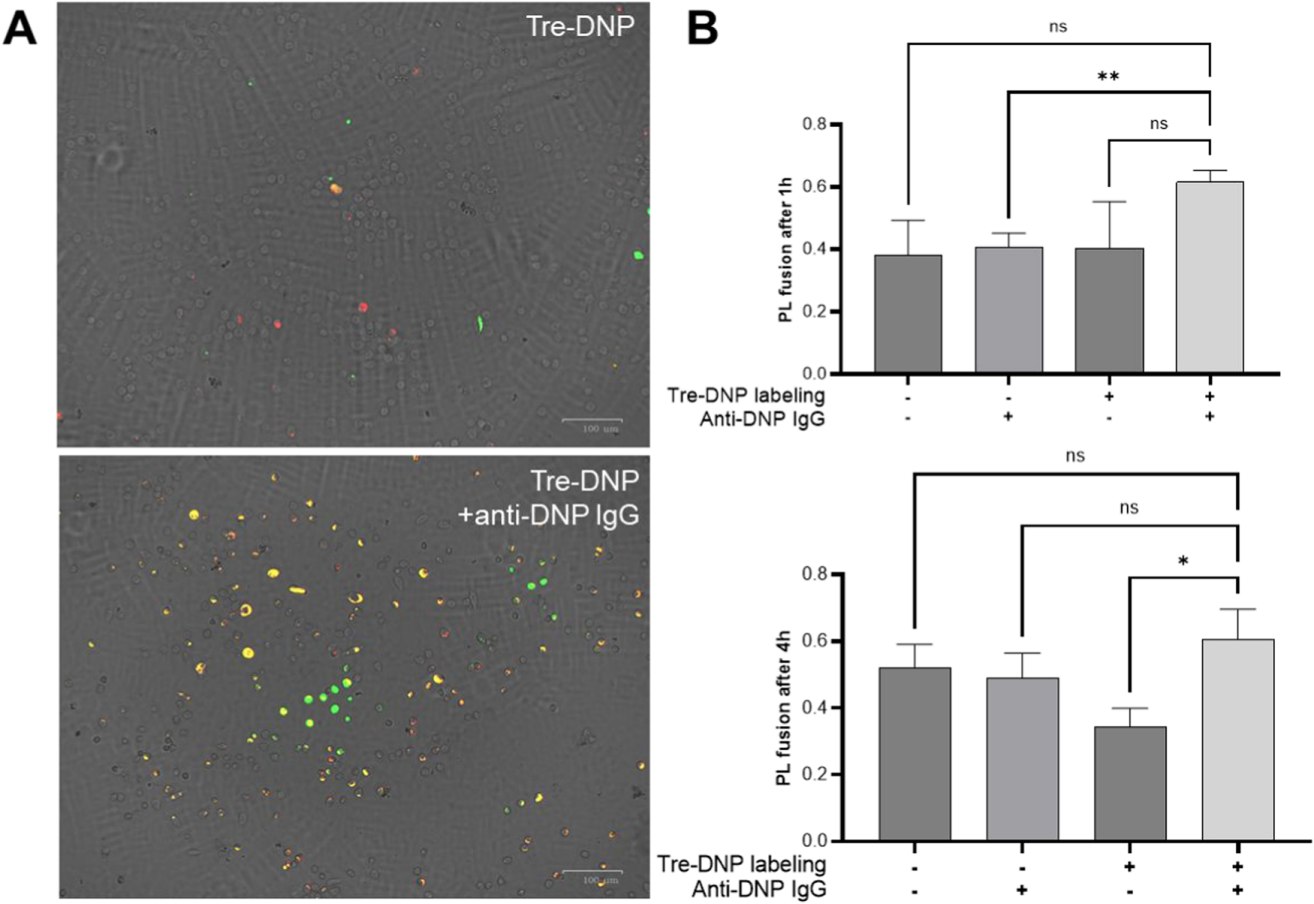
Tre-DNP increases phagolysosome fusion. Mabs was either labeled with Tre-DNP or left unlabeled and stained with 20 μM CMFDA (green). Bacteria were then incubated in buffered saline or opsonized with anti-DNP antibody. The bacteria (2 × 10^6^ CFU/mL) were cocultured with J774 murine macrophages (4 × 10^5^ cells/mL) for 1 h, and extracellular bacteria were eliminated with 100 μg/mL gentamicin. At each time point, cells were stained with 0.5 μM LysoTracker Deep Red (red) and imaged by fluorescence microscopy. (A) Shown are two representative merged images of macrophages 4 h post infection. Yellow depicts overlap between CMFDA and LysoTracker Deep Red (B, scale bar = 100 μM). (B) Colocalization of CMDFA^+^ bacteria with LysoTracker, indicating phagolysosome (PL) fusion, was quantified in ImageJ. Error bars represent the standard error of the mean from four independent experiments. Statistical analysis was performed using a repeated-measures one-way ANOVA with Dunnett’s multiple comparison test (** *p* ≤ 0.01, * *p* ≤ 0.05).

## Discussion

Infectious diseases caused by pathogenic mycobacteria, such as Mtb, Mabs, and M. avium, pose substantial therapeutic challenges. There is a need for alternatives to antibiotic therapy because these pathogens are intrinsically antibiotic-tolerant and have emerged in multidrug-resistant form, and new antibiotic options remain limited. As a potential tool to help address this challenge, we developed an immune targeting strategy for mycobacteria centered on the use of ARMs, which are small molecules that attach haptens onto pathogenic target cells, which subsequently recruit host-endogenous antibodies to promote immune clearance. ARMs have previously been developed for Gram-positive and Gram-negative bacteria using a variety of approaches (e.g., metabolic incorporation, antibiotic/aptamer-mediated incorporation) to deliver the ARM to bacteria-specific cell surface components (e.g., peptidoglycan or lipopolysaccharide).^30,33^ We previously developed the first ARM that specifically targets mycobacteria, Tre-DNP, for which proof-of-concept was established using the model organism M. smegmatis.^27^ Like other literature-reported trehalose-based mycobacteria metabolic labeling tools,^44^ Tre-DNP metabolically incorporates into surface glycolipids by exploiting a trehalose lipidation pathway that is evolutionarily conserved in mycobacterial species, but not present in humans or Gram-positive or Gram-negative bacteria.^44^ In principle, this conserved incorporation mechanism means that, in addition to M. smegmatis, the trehalose-based ARM strategy should be effective at targeting pathogenic mycobacteria.

The present study investigated the ability of Tre-DNP to label and promote antibody-mediated immune clearance of three mycobacterial pathogens with high human health relevance: Mtb, which causes TB disease and is responsible for over 1 million deaths annually; and NTM species, including Mabs and M. avium, which cause opportunistic respiratory illnesses of increasing prevalence. Evidence suggests that antibodies can protect the host against these pathogens. For example, the BCG vaccine-induced IgG antibodies enhanced phagocytosis and reduced Mtb growth.^25^ In addition, monoclonal antibodies targeting Mtb antigens like PstS1 and arabinomannan promoted bacterial clearance in an FcγR-dependent manner.^26^ Given that these antigens are prone to variation, we hypothesized that a mycobacteria ARM strategy would be effective against the pathogens tested, and specifically that Tre-DNP-modified, anti-DNP-opsonized mycobacteria would undergo enhanced uptake and killing by macrophages. In support of this hypothesis, here we found that Tre-DNP efficiently surface-labels and recruits anti-DNP antibodies to all species, and importantly that mycobacteria opsonized through this strategy were phagocytosed and eliminated at elevated levels compared to control conditions. One limitation of this study was the use of Mtb auxotroph, which are a safe model to work with but may not accurately reflect the dynamics of virulent strains and hindered our ability to use this strain for intracellular, mechanistic experiments. Although future experiments will aim to carry this work in vivo and to virulent Mtb, these data suggest that the ARM strategy should be broadly effective against all mycobacteria, while sparing the host and its beneficial microbiota. The Tre-DNP ARM strategy is designed to address some of the limitations of conventional antibody therapies, which are challenging to develop for mycobacteria.^60^ Mycobacteria can alter, mask, or downregulate the expression of target antigens, resulting in a heterogeneous pool of antibodies with varying affinities and effectiveness for pathogen recognition.^26,55,69,70^ In contrast, Tre-DNP labels mycobacteria by exploiting a conserved and essential biosynthetic pathway, and thus should not be vulnerable to such antigenic variations.

Transitioning from in vitro to in vivo studies presents future opportunities and challenges. In vivo models are essential for unraveling the immune mechanisms in a complex physiological environment and for evaluating the therapeutic efficacy and safety of ARMs as they progress toward clinical application. Although we envision ARMs therapeutically targeting mycobacteria in their brief extracellular period, several factors may impact the success of this approach in vivo, including the immune environment at the site of infection, physical barriers, bioavailability, and the enzymatic degredation.^71^ For example, the airway immune environment differs from the bloodstream with lower IgG concentrations^72^ that could limit the ability of Tre-DNP to effectively to enhance mycobacterial clearance. Additionally, several physical barriers in the airway,^73^ like respiratory epithelium, airway mucus, or pulmonary surfactants, could prevent Tre-DNP from reaching sufficient concentrations at the infection site. Thus, future studies should consider using targeted delivery systems, such as nebulization or intranasal delivery with mucolytic adjuvants,^73,74^ liposomal and nanoparticle carriers,^57^ intravenous administration, or a combination approaches to optimize ARM incorporation. Another important consideration is the stability of Tre-DNP in vivo. The presence of degradation enzymes such as esterases and/or trehalases could impact its stability, leading to premature metabolism, and potential of off-target effects. Enzyme-resistant linkages, such as amides and carbamates, can be considered for future ARM designs. Alternatively, a prodrug approach, where Tre-DNP is protected from sequestration (i.e., the capturing, isolation, premature degradation, or inactivation of the compound before it reaches its target) and activated only after reaching the intended target site could improve its efficacy. Finally, combination strategies that combine ARMs with antibiotics, vaccines, or monoclonal antibodies could potentiate antimicrobial effects. As one example, the exogenous hapten, rhamnose, can be added to vaccine formulations, driving increased anti- rhamnose immune responses.^75^ Similarly, Tre-DNP could be explored as a vaccine adjuvant,^75,76^ potentially reducing the need for antibiotics, as well as minimize the potential of developing drug resistance.^77,78^

Another advance of the present study was to examine the mechanism by which Tre-DNP increases phagocytosis and killing of pathogenic mycobacteria. Although a number of bacteria-specific ARMs have been developed and shown to promote uptake and/or killing of their target,^28–33^ to date there has not been a detailed mechanistic study of how these tools promote phagocytosis and killing by host macrophages. Here, we found that ARM-enhanced processing of bacteria by macrophages was driven by FcγR-mediated uptake and downstream increases in ROS production and PL fusion, consistent with the prior literature demonstrating antibody-mediated protection against mycobacteria discussed above.^25,26^ These findings offer insights into how the ARM amplifies the immune response against mycobacterial pathogens, suggesting a similar potential for other bacteria-targeting ARMs. Additional antimicrobial mechanisms may also contribute to mycobacteria killing, including increased production of antimicrobial peptides, modulation of nutritional immunity through altered sequestration of essential metals such as iron and zinc, and the induction of autophagy.^11^ In autophagy, a double membrane forms around damaged organelles or intracellular pathogens, generating an autophagosome that subsequently fuses lysosomes for destruction. This pathway has been shown to decrease the survival of mycobacteria, including NTM, by promoting their delivery to degradative compartments,^11^ and was induced in monocytes, macrophages, and dendritic cells in response to intravenous antibody treatment.^79^ Our results show that ARMs can be used as tools to interrogate the mechanistic details of the antibody-mediated host immune response to bacterial infection.

## Methods

### Mycobacterial Strains and Growth Conditions

The bacterial strains used in this work included Mtb mc^2^7000 (attenuated pantothenate auxotroph approved for use in Biosafety Level 2 containment),^48^ Mabs ATCC 19977, and M. avium mc^2^2500. Mabs *and* M. avium were cultured in Middlebrook 7H9 liquid medium supplemented with 10% v/v OADC (oleic acid, albumin, dextrose, and catalase), 0.5% v/v glycerol, and 0.05% v/v Tween-80. Mtb was cultured in the same 7H9 medium additionally supplemented with 100 μg/mL d-pantothenate. Bacteria were cultured at 37 °C in a shaking incubator. To initiate experiments, starter cultures grown to mid logarithmic phase with an optical density at 600 nm (OD_600_) of ∼0.5–1.2 were used.

### Labeling of Mycobacteria with Tre-DNP

Metabolic incorporation experiments were conducted essentially as previously reported.^27,80^ Starter cultures were diluted to the desired OD_600_ in the appropriate 7H9 liquid medium for initiating experiments. Evaluation of Tre-DNP incorporation into bacterial strains was performed in triplicate in sterile flat-bottom 96-well plates. First, starter culture was diluted with appropriate 7H9 liquid medium to a final OD_600_ of 0.2. Cells were mixed with Tre-DNP stock solution (in H_2_O), or appropriate 7H9 medium for untreated controls, to achieve the desired compound concentration (0–1 mM) and a final volume of 200 μL. The plate was incubated at 37 °C with shaking in a microplate reader (Tecan Infinite F200 or M200 PRO operated by Tecan iControl software) for 4, 12, and 24 h for Mabs, M. avium, and Mtb, respectively. Following the Tre-DNP incubation step, well contents were transferred to a v-bottom 96-well plate and the cells were centrifuged (3200*g*, 5 min, 4 °C) and washed with 1× phosphate-buffered saline containing 0.1% bovine serum albumin (PBS-B) three times. Cell pellets were resuspended in 99 μL of PBS. To each well, 1 μL of 1 mg/mL monoclonal mouse anti-DNP IgG antibodies (Millipore) (or 2 μL of 0.5 mg/mL mouse IgG1 kappa isotype control (Invitrogen)) was added to a final concentration of 10 μg/mL, followed by incubation at 37 °C for 1 h. Cells were washed three times with PBS-B and resuspended in 99 μL of PBS. To each well, 1 μL of 0.2 mg/mL antimouse IgG-PE (BioLegend) was added to a final concentration of 2 μg/mL for 30 min at 37 °C in the dark. Cells were washed three times with PBS-B and then fixed in 4% formaldehyde in PBS (180 μL) for 20 min at room temperature in the dark. Fixed cells were centrifuged and washed with PBS-B three times, resuspended in 100 μL of PBS, and then analyzed by flow cytometry.

### Flow Cytometry

After antibody staining of bacteria according to the above general procedure, 20 μL of the resuspended bacteria were transferred to 200 μL of PBS in a flat-bottom 96-well plate and analyzed by flow cytometry. Flow cytometry was performed on a CytoFlex flow cytometer (Beckman Coulter) or BD FACSAria III. Fluorescence data was collected at an event rate of 500–1000 events/s. ≥10,000 total events were collected. All flow cytometry experiments were performed with three replicate samples, and data shown were representative of at least two independent experiments. Scatter-gated fluorescence analysis was used to obtain mean fluorescence intensities.

### CMFDA Staining

Mycobacterial strains (Mabs, M. avium, and Mtb) were labeled with and without Tre-DNP and washed as described in the labeling procedure above. After washing, cells were stained with 5-chloromethylfluorescein diacetate (CMFDA; MedChemExpress).^81^ Tre-DNP-labeled and unlabeled bacteria were resuspended in 180 μL of a 10 μM working solution of CMFDA in PBS or PBS alone for unstained controls. The plate was incubated at 37 °C in the dark for 1 h. Following incubation, cells were centrifuged (3200*g*, 5 min, 4 °C) and washed twice with PBS-B.

To validate CMFDA staining, pellets were resuspended in 99 μL of PBS and treated with 1 μL of 1 mg/mL of anti-DNP IgG antibodies to a final concentration of 10 μg/mL. Samples were incubated at 37 °C for 1 h, washed three times with PBS-B. Next, cells were resuspended in 99 μL PBS and 1 μL of 0.2 mg/mL antimouse IgG-PE (BioLegend) was added to each well to achieve a final concentration of 2 μg/mL. Cells were incubated at 37 °C in the dark for 30 min, washed three times with PBS-B, and fixed in 180 μL of 4% formaldehyde in PBS for 20 min at room temperature. Fixed cells were centrifuged, washed three times with PBS-B, and resuspended for flow cytometry analysis as above.

### Phagocytosis Assay

THP-1 cells were purchased from ATCC. Cells were maintained between 2 × 10^5^ and 10 × 10^5^ cells/mL in RPMI I640 (Gibco) supplemented with 10% v/v heat-inactivated fetal bovine serum, 0.05 mM 2-mercaptoethanol, 1% v/v penicillin-streptomycin and incubated at 37 °C and in 5% CO_2_. Cells were used between passage numbers 5 and 20. THP-1 cells were seeded in complete media at 1 × 10^6^ cells/mL into 12-well treated tissue culture plates and differentiated with 100 nM PMA in complete media for 16 h in 5% CO_2_ at 37 °C. Media was replaced, and cells were allowed to rest for 48 h.

Before the phagocytosis assay, bacterial species were labeled with Tre-DNP or left untreated and then stained with CMFDA with concentrations as described in previous procedures. Cells were mixed with Tre-DNP stock solution (in H_2_O) or appropriate 7H9 medium for untreated controls to achieve the desired compound concentration (250 μM) and a final volume of 1.0 mL in a 12-well flat-bottom plate. The plate was incubated at 37 °C with shaking at 220 rpm for 4, 12, and 24 h for Mabs, M. avium, and Mtb, respectively. Cells were then transferred to 1.5 mL microcentrifuge tubes and centrifuged (3200*g*, 5 min, 4 °C). Cells were washed twice with HBSS. The pellet was resuspended in 1.0 mL of HBSS, and the OD600 was taken, followed by calculations for 1 × 10^7^ CFU/mL. Volumes were transferred into a 1.5 mL centrifuge tube and pelleted by centrifugation (3,200*g*, 5 min, 4 °C). The pellet was resuspended in 1.0 mL of 10 μM CMFDA in PBS and incubated for 1 h at 37 °C in the dark. The stained bacterial cells were washed twice with 1 mL of PBS-B and then used for phagocytosis. The CMFDA-stained, Tre-DNP modified and unmodified bacteria were subjected to either mock treatment or antibody opsonization with anti-DNP IgG antibodies at concentrations described in the labeling procedure. Cells were washed with PBS-B and resuspended in 1.0 mL phagocytosis buffer (5 mM CaCl2 in RPMI). PMA-differentiated THP-1 cells were cocultured with bacteria at a 1:10 MOI. To synchronize phagocytosis, cells were centrifuged at 1600 rpm for 5 min at 4 °C and incubated for 1 h. Subsequently, THP-1 cells were washed three times with 1 mL RPMI and harvested by trypsinization. Cells were washed in FACS buffer (0.1% BSA in PBS), fixed with 2% formaldehyde in PBS for 20 min, washed three times, and stained with 5 μL of antihuman CD14-APC (BioLegend) per million cells for 20 min. Cells were again washed twice and resuspended in PBS for flow cytometry. To determine the role of FcγR in phagocytosis, differentiated THP-1 cells were preincubated for 30 min with a 1:4 dilution of human Fc receptor binding inhibitor antibody (Thermo Fisher Scientific) before coculturing with the bacteria.

### Killing Assay

Killing of Mabs 19977 and M. avium mc^2^2500 were determined using a CFU assay. For this assay, Tre-DNP modified or nonmodified mycobacteria were subjected to mock treatment or opsonized with anti-DNP IgG and were cocultured with 2.0 × 10^5^ differentiated THP-1 (in a cell-treated 96 well plate) at an MOI of 10 for 1 h at 37 °C in 5% CO_2_. After infection, the cells were washed extensively with complete media and left in media containing 200 μg/mL of amikacin (but no other antibiotic) for 1 h to kill any extracellular bacteria. After 1 h, the amikacin was lowered to 50 μg/mL and at indicated time points, THP-1 cells were lysed with 0.05% v/v tween-20 in ultrapure water and diluted with 0.1% Tween 80 in 0.9% saline. Serial dilutions of lysates were plated in triplicate on 7H10 agar and incubated at 37 °C. CFU were enumerated after 3 days *Mabs WT* or 7 days for *M*. *avium* mc^2^2500. The replication index was calculated as CFU obtained at each time point (*t*) after infection/CFU obtained at the initial time point after infection.

### Alamar Blue Viability Assay

Under the same conditions as the killing assay, THP-1 cell viability was quantified by Alamar blue assay.^82^ For each condition, cells were incubated with 10% v/v of Alamar Blue solution for 4 h at 37 °C in 5% CO_2_. The fluorescence was measured in a fluorescent plate reader (Tecan Infinite F200 or M200 PRO operated by Tecan iControl software) at an excitation wavelength of 560 nm and an emission wavelength of 590 nm. Cell viability was determined by normalizing the fluorescence values of infected THP-1 wells to those of uninfected controls, and expressed as a percentage of viability.

### ROS Quantification Assay

ROS production was assessed using the cell-permeant ROS indicator H_2_DCFDA (MedChemExpress), which fluoresces after oxidation by superoxide products. The assay was performed as described here^83^ with the following modifications: differentiated THP-1 cells (2.0 × 10^5^) were plated in triplicate in a 96-well black polystyrene flat bottom plate. For blocking FcγR, the differentiated THP-1 cells were preincubated for 30 min with 1:4 dilution of human Fc receptor binding inhibitor Ab (ThermoFisher Scientific) before coculturing with the bacteria.^56^ THP-1 cells were then treated with modified Mabs WT or M. avium mc^2^2500, followed by an amikacin protection assay similar to the Killing assay. At indicated time points, cells were treated with 10 μM H_2_DCFDA for 15 min at 37 °C in the dark. Cells were washed three times with PBS, and fluorescence was measured in the plate reader at an excitation wavelength of 488 nm and an emission wavelength of 525 nm.^57^

### Phagosome-Lysosome Fusion Assay

J774 cells were purchased from ATCC and maintained in DMEM supplemented with 10% fetal bovine serum (FBS) and 1% penicillin-streptomycin at 37 °C in a 5% CO_2_. For the assay, cells were seeded onto 18 mm glass coverslips in a 12 well plate at a density of 4 × 10^5^ cells per well. Bacteria were labeled with Tre-DNP or left unmodified and opsonized with anti-DNP IgG (or left alone). J774 macrophages were cocultured with bacteria at a 1:5 MOI for 1 h and after washing, media was replaced with media containing 100 μg/mL gentamicin to eliminate extracellular bacteria. At 1 and 4 h, LysoTracker Deep Red (250 nM final concentration, ThermoFisher Scientific) was added to the wells and incubated at 37 °C for 6–10 min. Cells were subsequently washed with PBS to remove excess dye, and coverslips were mounted onto slides using Vectashield antifade mounting medium (VectorLabs). Images were acquired at 63× magnification the Zoe Fluorescent Cell Imager (BioRad Laboratories), and to assess phagolysosome fusion, the colocalization of CMFDA-labeled bacteria with LysoTracker was quantified using Manders’ coefficient in image J with the JACoP plug-in.^84^

## Supplementary Material



## Notes

Representative flow cytometry dot plots for CMFDA staining experiment; CMFDA stains M. avium without affecting Tre-DNP labeling; representative flow cytometry contour plots and gating strategy for phagocytosis experiments; intracellular killing of mycobacteria was not due to THP-1 cell death; representative images for phagolysosome fusion experiments (PDF)

## References

[1] World Health Organization . Global Tuberculosis Report 2024 https://www.who.int/teams/global-programme-on-tuberculosis-and-lung-health/tb-reports/global-tuberculosis-report-2024.

[2] Winthrop K. L., Marras T. K., Adjemian J., Zhang H., Wang P., Zhang Q. (2020). Incidence and Prevalence
of Nontuberculous Mycobacterial Lung Disease
in a Large U. S. Managed Care Health Plan, 2008 – 2015. Ann. Am. Thorac. Soc..

[3] Dahl V. N., Mølhave M., Fløe A., van Ingen J., Schön T., Lillebaek T., Andersen A. B., Wejse C. (2022). Global Trends
of Pulmonary Infections with Nontuberculous Mycobacteria: A Systematic
Review. Int. J. Infect. Dis..

[4] Ramsay L. C., Shing E., Wang J., Marras T. K., Kwong J. C., Brode S. K., Jamieson F. B., Sander B. (2020). Costs Associated with
Nontuberculous Mycobacteria Infection, Ontario, Canada, 2001–2012. Emerging Infect. Dis..

[5] Ratnatunga C. N., Lutzky V. P., Kupz A., Doolan D. L., Reid D. W., Field M., Bell S. C., Thomson R. M., Miles J. J. (2020). The Rise
of Non-Tuberculosis Mycobacterial Lung Disease. Front. Immunol..

[6] Sousa S., Bandeira M., Carvalho P. A., Duarte A., Jordao L. (2015). Nontuberculous
Mycobacteria Pathogenesis and Biofilm Assembly. Int. J. Mycobacteriol..

[7] Nasiri M. J., Haeili M., Ghazi M., Goudarzi H., Pormohammad A., Fooladi A. A. I., Feizabadi M. M. (2017). New Insights
in to the Intrinsic
and Acquired Drug Resistance Mechanisms in Mycobacteria. Front. Microbiol..

[8] Emane A. K. A., Guo X., Takiff H. E., Liu S. (2021). Drug Resistance, Fitness
and Compensatory Mutations in Mycobacterium Tuberculosis. Tuberculosis.

[9] Gill C. M., Dolan L., Piggott L. M., McLaughlin A. M. (2022). New Developments
in Tuberculosis Diagnosis and Treatment. Breathe.

[10] Kumar K., Daley C. L., Griffith D. E., Loebinger M. R. (2022). Management
of Mycobacterium Avium Complex and Mycobacterium Abscessus Pulmonary
Disease: Therapeutic Advances and Emerging Treatments. Eur. Respir. Rev..

[11] Awuh J. A., Flo T. H. (2017). Molecular Basis
of Mycobacterial Survival in Macrophages. Cell.
Mol. Life Sci..

[12] Gopalaswamy R., Shanmugam S., Mondal R., Subbian S. (2020). Of Tuberculosis and
Non-Tuberculous Mycobacterial Infections - A Comparative Analysis
of Epidemiology, Diagnosis and Treatment. J.
Biomed. Sci..

[13] Daher W., Pichler V., Karam J., Neyrolles O., Kremer L. (2023). The Molecular Basis and Downstream
Immune Consequences
of Mycobacteria-Host Cell Interactions. FEMS
Microbiol. Rev..

[14] Weiss G., Schaible U. E. (2015). Macrophage Defense Mechanisms against Intracellular
Bacteria. Immunol. Rev..

[15] Rhoades E. R., Archambault A. S., Greendyke R., Hsu F.-F., Streeter C., Byrd T. F. (2009). Mycobacterium
Abscessus Glycopeptidolipids Mask Underlying
Cell Wall Phosphatidyl- Myo -Inositol Mannosides Blocking Induction
of Human Macrophage TNF-α by Preventing Interaction with TLR2. J. Immunol..

[16] Fratti R. A., Chua J., Vergne I., Deretic V. (2003). Mycobacterium Tuberculosis
Glycosylated Phosphatidylinositol Causes Phagosome Maturation Arrest. Proc. Natl. Acad. Sci. U.S.A..

[17] Kang P. B., Azad A. K., Torrelles J. B., Kaufman T. M., Beharka A., Tibesar E., DesJardin L. E., Schlesinger L. S. (2005). The Human
Macrophage Mannose Receptor Directs Mycobacterium Tuberculosis Lipoarabinomannan-Mediated
Phagosome Biogenesis. J. Exp. Med..

[18] Nguyen L., Pieters J. (2005). The Trojan Horse: Survival Tactics of Pathogenic Mycobacteria
in Macrophages. Trends Cell Biol..

[19] Matsuyama M., Hizawa N., Nonaka M., Nakajima M., Morishima Y., Ishii Y. (2021). The Role of Nrf2 in
Mycobacterial Infection. Antioxidants.

[20] Sia J. K., Rengarajan J. (2019). Immunology
of Mycobacterium Infections. Microbiol. Spectrum.

[21] Lu L. L., Chung A. W., Rosebrock T. R., Ghebremichael M., Yu W. H., Grace P. S., Schoen M. K., Tafesse F., Martin C., Leung V., Mahan A. E., Sips M., Kumar M. P., Tedesco J., Robinson H., Tkachenko E., Draghi M., Freedberg K. J., Streeck H., Suscovich T. J., Lauffenburger D. A., Restrepo B. I., Day C., Fortune S. M., Alter G. (2016). A Functional
Role for Antibodies in Tuberculosis. Cell.

[22] Li H., Javid B. (2018). Antibodies and Tuberculosis:
Finally Coming of Age?. Nat. Rev. Immunol..

[23] Rijnink W. F., Ottenhoff T. H. M., Joosten S. A. (2021). B-Cells and Antibodies as Contributors
to Effector Immune Responses in Tuberculosis. Front. Immunol..

[24] Hermann C., King C. G. (2021). TB or Not to Be:
What Specificities and Impact Do Antibodies
Have during Tuberculosis?. Oxford Open Immunol..

[25] De
Vallière S., Abate G., Blazevic A., Heuertz R. M., Hoft D. F. (2005). Enhancement of Innate and Cell-Mediated Immunity by
Antimycobacterial Antibodies. Infect. Immun..

[26] Watson A., Li H., Ma B., Weiss R., Bendayan D., Abramovitz L., Ben-Shalom N., Mor M., Pinko E., Bar Oz M., Wang Z., Du F., Lu Y., Rybniker J., Dahan R., Huang H., Barkan D., Xiang Y., Javid B., Freund N. T. (2021). Human Antibodies Targeting a Mycobacterium
Transporter Protein Mediate Protection against Tuberculosis. Nat. Commun..

[27] Dzigba P., Rylski A. K., Angera I. J., Banahene N., Kavunja H. W., Greenlee-Wacker M. C., Swarts B. M. (2023). Immune Targeting
of Mycobacteria
through Cell Surface Glycan Engineering. ACS
Chem. Biol..

[28] Spiegel D. A. (2013). A Call
to ARMs: The Promise of Immunomodulatory Small Molecules. Expert Rev. Clin. Pharmacol..

[29] McEnaney, P. J. ; Parker, C. G. ; Zhang, A. X. Antibody-Recruiting Small Molecules: Synthetic Constructs as Immunotherapeutics. In Annual Reports in Medicinal Chemistry; Elsevier, 2017; Vol. 50, pp 481–518.

[30] Feigman M. J. S., Pires M. M. (2018). Synthetic Immunobiotics: A Future Success Story in
Small Molecule-Based Immunotherapy?. ACS Infect.
Dis..

[31] Ghai T., Das A., Patel R. (2022). The Investigation of
the Effect of Antibody Recruiting
on Various Antigenic Markers (Cancer, Bacteria, Viruses): A Literature
Review. Undergrad. Res. Nat. Clin. Sci. Technol.
J..

[32] Charles W. Z., Faries C. R., Street Y. T., Flowers L. S., McNaughton B. R. (2022). Antibody-Recruitment
as a Therapeutic Strategy: A Brief History and Recent Advances. ChemBioChem.

[33] Dzigba P., Seth M. A., Greenlee-Wacker M. C., Swarts B. M. (2025). Redirecting the
Host Immune Response to Bacterial Infection with Antibody-Recruiting
Molecules (ARMs), Current Opinion in Chemical Biology. Curr. Opin. Chem. Biol..

[34] Zhang A. X., Murelli R. P., Barinka C., Michel J., Cocleaza A., Jorgensen W. L., Lubkowski J., Spiegel D. A. (2010). A Remote Arene-Binding
Site on Prostate Specific Membrane Antigen Revealed by Antibody-Recruiting
Small Molecules. J. Am. Chem. Soc..

[35] Küchenthal C., Maison W. (2010). Antibody Recruiting Small Molecules: A New Option for
Prostate Tumor Therapy by PSMA Targeting. ChemBioChem.

[36] Rullo A. F., Fitzgerald K. J., Muthusamy V., Liu M., Yuan C., Huang M., Kim M., Cho A. E., Spiegel D. A. (2016). Re-Engineering
the Immune Response to Metastatic Cancer: Antibody-Recruiting Small
Molecules Targeting the Urokinase Receptor. Angew. Chem., Int. Ed..

[37] Parker C. G., Domaoal R. A., Anderson K. S., Spiegel D. A. (2009). An Antibody-Recruiting
Small Molecule That Targets HIV Gp120. J. Am.
Chem. Soc..

[38] Chirkin E., Muthusamy V., Mann P., Roemer T., Nantermet P. G., Spiegel D. A. (2017). NeutralizationofPathogenicFungiwith Small-MoleculeImmunotherapeutics. Angew. Chem., Int. Ed..

[39] Kaewsapsak P., Esonu O., Dube D. H. (2013). Recruiting
the Host’s Immune
System To Target Helicobacter Pylori’s Surface Glycans. ChemBioChem.

[40] Fura J. M., Pires M. M. (2015). D-Amino Carboxamide-Based Recruitment of Dinitrophenol
Antibodies to Bacterial Surfaces via Peptidoglycan Remodeling. Biopolymers.

[41] Sabulski M. J., Pidgeon S. E., Pires M. M. (2017). Immuno-Targeting of Staphylococcus
Aureus via Surface Remodeling Complexes. Chem.
Sci..

[42] Hyun J. Y., Lee C. H., Lee H., Jang W. D., Shin I. (2020). Bacterial
Lectin-Targeting Glycoconjugates for Selective Elimination of Pathogenic
Bacteria. ACS Macro Lett..

[43] Dalesandro B. E., Pires M. M. (2023). Immunotargeting
of Gram-Positive Pathogens via a Cell
Wall Binding Tick Antifreeze Protein. J. Med.
Chem..

[44] Banahene N., Kavunja H. W., Swarts B. M. (2022). Chemical Reporters
for Bacterial
Glycans: Development and Applications. Chem.
Rev..

[45] Ortega E., Kostovetzky M., Larralde C. (1984). Natural DNP-Binding
Immunoglobulins
and Antibody Multispecificity. Mol. Immunol..

[46] Jakobsche C. E., Parker C. G., Tao R. N., Kolesnikova M. D., Douglass E. F., Spiegel D. A. (2013). Exploring Binding and Effector Functions
of Natural Human Antibodies Using Synthetic Immunomodulators. ACS Chem. Biol..

[47] Dautin N., de Sousa-d’Auria C., Constantinesco-Becker F., Labarre C., Oberto J., de la Sierra-Gallay I. L., Dietrich C., Issa H., Houssin C., Bayan N. (2017). Mycoloyltransferases:
A Large and Major Family of Enzymes Shaping the Cell Envelope of Corynebacteriales. Biochim. Biophys. Acta, Gen. Subj..

[48] Sambandamurthy V. K., Derrick S. C., Hsu T., Chen B., Larsen M. H., Jalapathy K. V., Chen M., Kim J., Porcelli S. A., Chan J., Morris S. L., Jacobs W. R. (2006). Mycobacterium Tuberculosis
ΔRD1 ΔpanCD: A Safe and Limited Replicating Mutant Strain
That Protects Immunocompetent and Immunocompromised Mice against Experimental
Tuberculosis. Vaccine.

[49] Huygen K. (2014). The Immunodominant
T-Cell Epitopes of the Mycolyl-Transferases of the Antigen 85 Complex
of M. Tuberculosis. Front. Immunol..

[50] Daffé M., Marrakchi H. (2019). Unraveling the Structure of the Mycobacterial Envelope. Microbiol. Spectrum.

[51] Breeuwer P., Drocourt J.-L., Rombouts F. M., Abee T. (1994). Energy-Dependent, Carrier-Mediated
Extrusion of Carboxyfluorescein from Saccharomyces Cerevisiae Allows
Rapid Assessment of Cell Viability by Flow Cytometry. Appl. Environ. Microbiol..

[52] Jimenez-Duran G., Luque-Martin R., Patel M., Koppe E., Bernard S., Sharp C., Buchan N., Rea C., de Winther M. P. J., Turan N., Angell D., Wells C. A., Cousins R., Mander P. K., Masters S. L. (2020). Pharmacological Validation of Targets
Regulating CD14 during Macrophage Differentiation. EBioMedicine.

[53] Aderem A., Underhill D. M. (1999). Mechanisms of Phagocytosis in Macrophages. Annu. Rev. Immunol..

[54] Stokes R. W., Doxsee D. (1999). Receptor-Mediated Uptake,
Survival, Replication, and
Drug Sensitivity of Mycobacterium Tuberculosis within the Macrophage-like
Cell Line THP-1: A Comparison with Human Monocyte-Derived Macrophages. Cell. Immunol..

[55] Liu Y., Chen T., Zhu Y., Furey A., Lowary T. L., Chan J., Bournazos S., Ravetch J. V., Achkar J. M. (2023). Features
and Protective Efficacy of Human Monoclonal Antibodies Targeting Mycobacterium
Tuberculosis Arabinomannan. JCI Insight.

[56] Grasse M., Rosenkrands I., Olsen A., Follmann F., Dietrich J. (2018). A Flow Cytometry-Based
Assay to Determine the Phagocytic Activity of Both Clinical and Nonclinical
Antibody Samples against Chlamydia Trachomatis. Cytometry, Part A.

[57] Poerio N., Riva C., Olimpieri T., Rossi M., Lorè N. I., De Santis F., De Angelis L. H., Ciciriello F., D’andrea M. M., Lucidi V., Cirillo D. M., Fraziano M. (2022). Combined Host-and
Pathogen-Directed Therapy for the Control of Mycobacterium Abscessus
Infection. Microbiol. Spectrum.

[58] Kim J.-H., Chaurasia A. K., Batool N., Ko K. S., Kim K. K. (2019). Alternative
Enzyme Protection Assay To Overcome the Drawbacks of the Gentamicin
Protection Assay for Measuring Entry and Intracellular Survival of
Staphylococci. Infect. Immun..

[59] Kumar S. K., Singh P., Sinha S. (2015). Naturally
Produced Opsonizing Antibodies
Restrict the Survival of Mycobacterium Tuberculosis in Human Macrophages
by Augmenting Phagosome Maturation. Open Biol..

[60] Bournazos S., Gupta A., Ravetch J. V. (2020). The Role of IgG Fc Receptors in Antibody-Dependent
Enhancement. Nat. Rev. Immunol..

[61] Canton M., Sánchez-Rodríguez R., Spera I., Venegas F. C., Favia M., Viola A., Castegna A. (2021). Reactive Oxygen Species
in Macrophages: Sources and Targets. Front.
Immunol..

[62] Moghadam Z. M., Henneke P., Kolter J. (2021). From Flies
to Men: ROS and the NADPH
Oxidase in Phagocytes. Front. Cell Dev. Biol..

[63] Dupré-Crochet S., Erard M., Nüβe O. (2013). ROS Production
in Phagocytes: Why,
When, and Where?. J. Leukocyte Biol..

[64] Bermudez L. E. M., Young L. S. (1989). Oxidative and Non-Oxidative Intracellular Killing of
Mycobacterium Avium Complex. Microb. Pathog..

[65] Gomes M. S., Appelberg R. (2002). NRAMP1-or
Cytokine-Induced Bacteriostasis of Mycobacterium
Avium by Mouse Macrophages Is Independent of the Respiratory Burst. Microbiology.

[66] de
Chastellier C., Thilo L. (2002). Pathogenic Mycobacterium Avium Remodels
the Phagosome Membrane in Macrophages within Days after Infection. Eur. J. Cell Biol..

[67] Joller N., Weber S. S., Müller A. J., Spörri R., Selchow P., Sander P., Hilbi H., Oxenius A. (2010). Antibodies
Protect against Intracellular Bacteria by Fc Receptor-Mediated Lysosomal
Targeting. Proc. Natl. Acad. Sci. U.S.A..

[68] Henry R. M., Hoppe A. D., Joshi N., Swanson J. A. (2004). The Uniformity of
Phagosome Maturation in Macrophages. J. Cell
Biol..

[69] Mosa A. I. (2020). Antigenic
Variability. Front. Immunol..

[70] Van
Der Woude M. W., Bäumler A. J. (2004). Phase and Antigenic Variation in
Bacteria. Clin. Microbiol. Rev..

[71] Wu K., Kwon S. H., Zhou X., Fuller C., Wang X., Vadgama J., Wu Y. (2024). Overcoming
Challenges in Small-Molecule
Drug Bioavailability: A Review of Key Factors and Approaches. Int. J. Mol. Sci..

[72] Walsh L. J., Sullivan A., Ward C., Hunt E. B., Lapthorne S., Eustace J. A., Fanning L. J., Plant B. J., O’Byrne P. M., MacSharry J. A., Murphy D. M. (2024). Airway and Systemic Immunoglobulin
Profiling and Immune Response in Adult Asthma. Lung.

[73] Murgia X., De Souza Carvalho C., Lehr C. M. (2014). Overcoming the Pulmonary
Barrier:
New Insights to Improve the Efficiency of Inhaled Therapeutics. Eur. J. Nanomed..

[74] Plaunt A. J., Nguyen T. L., Corboz M. R., Malinin V. S., Cipolla D. C. (2022). Strategies
to Overcome Biological Barriers Associated with Pulmonary Drug Delivery. Pharmaceutics.

[75] Hossain M. K., Vartak A., Karmakar P., Sucheck S. J., Wall K. A. (2018). Augmenting
Vaccine Immunogenicity through the Use of Natural Human Anti-Rhamnose
Antibodies. ACS Chem. Biol..

[76] Tagliabue A., Rappuoli R. (2018). Changing Priorities
in Vaccinology: Antibiotic Resistance
Moving to the Top. Front. Immunol..

[77] Kilinç G., Saris A., Ottenhoff T. H. M., Haks M. C. (2021). Host-Directed Therapy
to Combat Mycobacterial Infections. Immunol.
Rev..

[78] Jeong E.-K., Lee H.-J., Jung Y.-J. (2022). Host-Directed Therapies for Tuberculosis. Pathogens.

[79] Das M., Karnam A., Stephen-Victor E., Gilardin L., Bhatt B., Sharma V. K., Rambabu N., Patil V., Lecerf M., Käsermann F., Bruneval P., Balaji K. N., Benveniste O., Kaveri S. V., Bayry J. (2020). Intravenous Immunoglobulin Mediates
Anti-Inflammatory Effects in Peripheral Blood Mononuclear Cells by
Inducing Autophagy. Cell Death Dis..

[80] Banahene, N. ; Swarts, B. M. Metabolic Labeling of Live Mycobacteria with Trehalose-Based Probes. In Methods in Molecular Biology; Springer, 2021; Vol. 2314, pp 385–398. 34235664 10.1007/978-1-0716-1460-0_18

[81] Purdy G. E., Niederweis M., Russell D. G. (2009). Decreased Outer Membrane Permeability
Protects Mycobacteria from Killing by Ubiquitin-Derived Peptides. Mol. Microbiol..

[82] Rampersad S. N. (2012). Multiple
Applications of Alamar Blue as an Indicator of Metabolic Function
and Cellular Health in Cell Viability Bioassays. Sensors.

[83] Banskota S., Wang H., Kwon Y. H., Gautam J., Haq S., Grondin J., Steinberg G. R., Khan W. I. (2023). Inhibition of NADPH
Oxidase (NOX) 2 Mitigates Colitis in
Mice with Impaired Macrophage AMPK Function. Biomedicines.

[84] Bolte S., Cordelières F.
P. (2006). A Guided Tour into Subcellular
Colocalization
Analysis in Light Microscopy. J. Microsc..

