# Supplementary material for: An Antibody-Recruiting Molecule Enhances Fcγ Receptor-Mediated Uptake and Killing of Mycobacterial Pathogens by Macrophages
Source: ACS Infect Dis. 2025 May 1;11(6):1563–76. doi: 10.1021/acsinfecdis.5c00097 (PMC12191616; doi:10.1021/acsinfecdis.5c00097)
Supplement: Supplementary file 1 [file id5c00097_si_001.pdf]

Supporting information (SI)

for:

An Antibody-Recruiting Molecule Enhances Fcγ Receptor-Mediated Uptake and Killing of Mycobacterial Pathogens by Macrophages

Priscilla Dzigba,[a,b,c] Grisha A.T. Dekhtyar,[e] Mary Jane Hartman,[d] Kai J. Winstead-Leroy,[e] Mallary C. Greenlee-Wacker[b,d]* and Benjamin M. Swarts[a,c]*

[a]Department of Chemistry and Biochemistry and [b]Department of Biology, Central Michigan University, Mount Pleasant, MI, 48859, USA

[c]Biochemistry, Cell, and Molecular Biology Graduate Programs, Central Michigan University, Mount Pleasant, MI, 48859 United States

[d]Biological Sciences Department and [e]Chemistry and Biochemistry Department, California Polytechnic State University, San Luis Obispo, CA, 93407, USA

*Corresponding author: E-mail: mcgreenl@calpoly.edu

*Corresponding author: E-mail: ben.swarts@cmich.edu

SI Table of Contents

I. Supplementary figures and schemes ______ S3
Figure S1. Representative flow cytometry dot plots for CMFDA staining experiment ______ S3
Figure S2. CMFDA stains *M. avium* without affecting Tre-DNP labeling ______ S4
Figure S3. CMFDA stains Mtb without affecting Tre-DNP labeling ______ S5
Figure S4. Representative flow cytometry contour plots and gating strategy for phagocytosis experiment ______ S6
Figure S5. Representative flow cytometry contour plots and gating strategy for FcγR-dependent phagocytosis experiments ______ S7
Figure S6. Intracellular killing of mycobacteria was not due to THP-1 cell death ______ S8
Figure S7. Representative images for phagolysosome fusion experiments ______ S9

I. Supplementary Figures

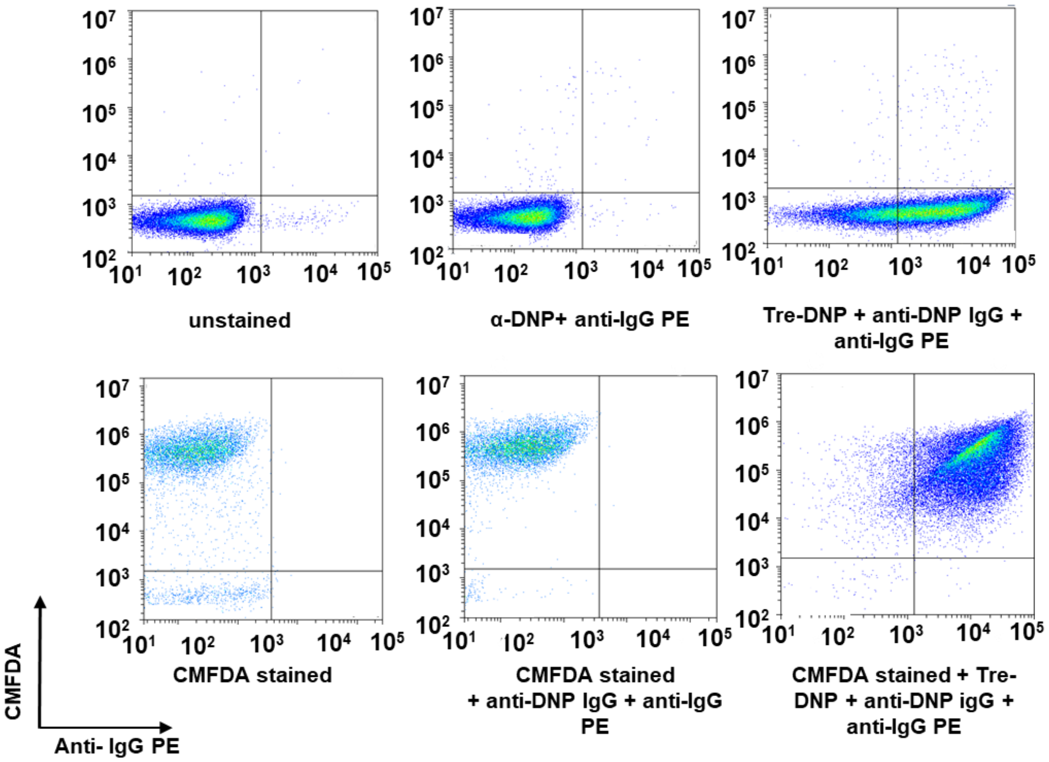

Figure S1. Representative flow cytometry dot plots for CMFDA staining experiment. Mabs was incubated with Tre-DNP (250 μM), then washed and stained with CMFDA (10 μM). Bacteria were washed, treated with mouse anti-DNP antibody (or mouse isotype control antibody), washed, and stained with PE-conjugated rabbit anti-mouse IgG antibody. Stained cells were fixed, washed, and analyzed by flow cytometry.

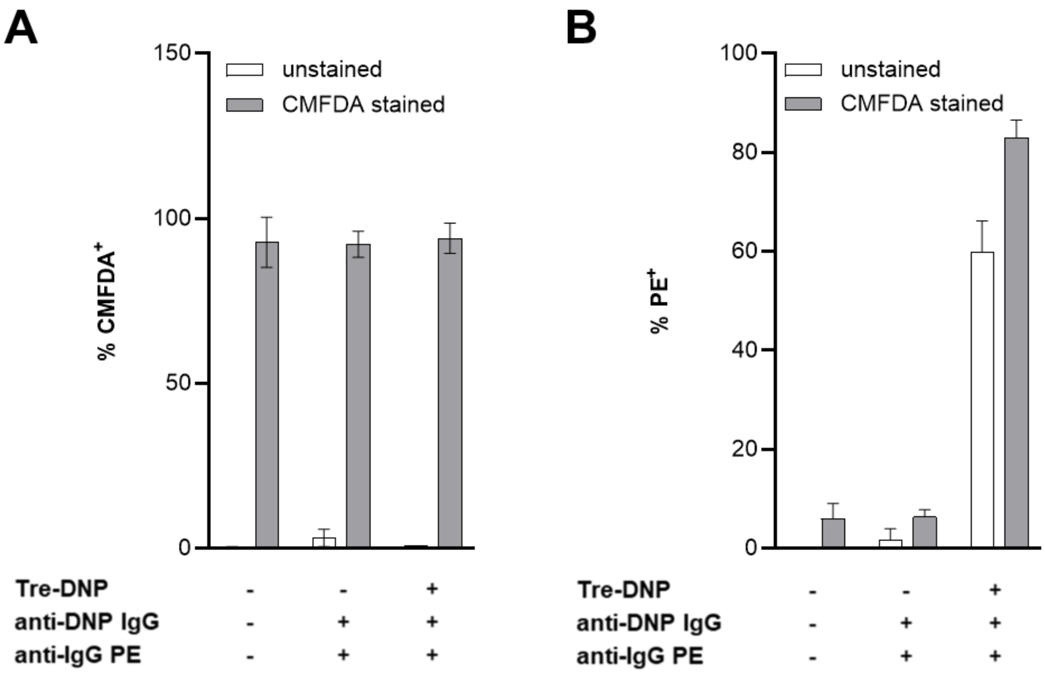

Figure S2. CMFDA stains *M. avium* without affecting Tre-DNP labeling. *M. avium* was labeled with Tre-DNP (250 μM) (or left unlabeled), then washed and stained with CMFDA (10 μM) (or left unstained). Bacteria were washed, treated with mouse anti-DNP antibody (or left untreated), washed, and stained with PE-conjugated rabbit anti-mouse IgG antibody. Stained cells were fixed, washed, and analyzed by flow cytometry. (A) Percentage of the population positive for fluorescein ($CMFDA^+$), representing CMFDA-stained cells. (B) Percentage of the population positive for PE (PE^+), representing antibody-bound cells. Data are presented as means with error bars denoting standard deviation of three replicate experiments.

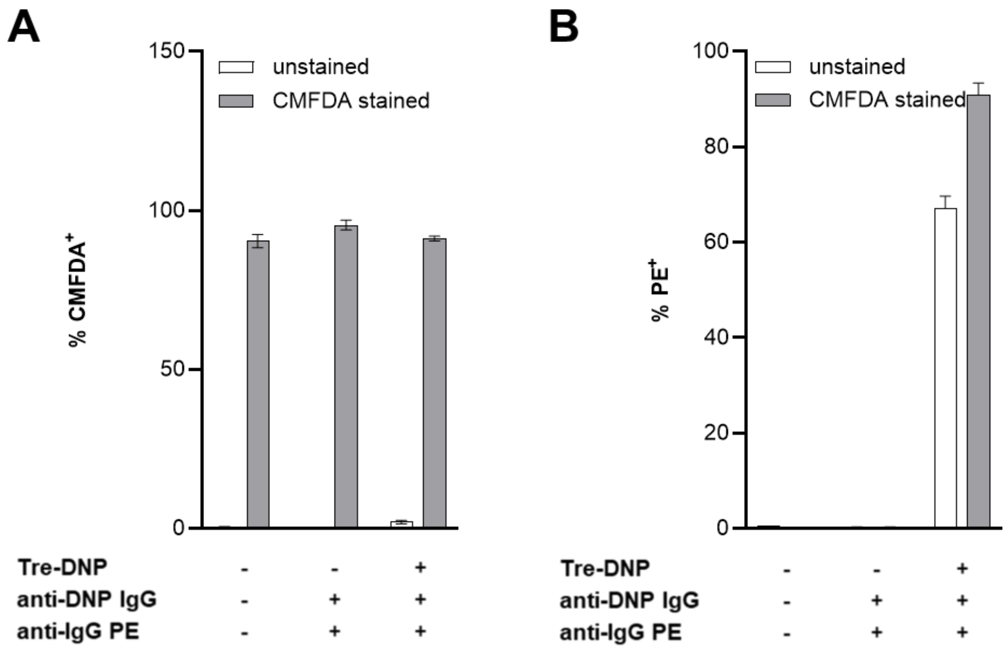

Figure S3. CMFDA stains Mtb without affecting Tre-DNP labeling. Mtb mc^{2}7000 was labeled with Tre-DNP (250 µM) (or left unlabeled), then washed and stained with CMFDA (10 µM) (or left unstained). Bacteria were washed, treated with mouse anti-DNP antibody (or left untreated), washed, and stained with PE-conjugated rabbit anti-mouse IgG antibody. Stained cells were fixed, washed, and analyzed by flow cytometry. (A) Percentage of the population positive for fluorescein (CMFDA$^+$), representing CMFDA-stained cells. (B) Percentage of the population positive for PE (PE$^+$), representing antibody-bound cells. Data are presented as means with error bars denoting standard deviation of three replicate experiments

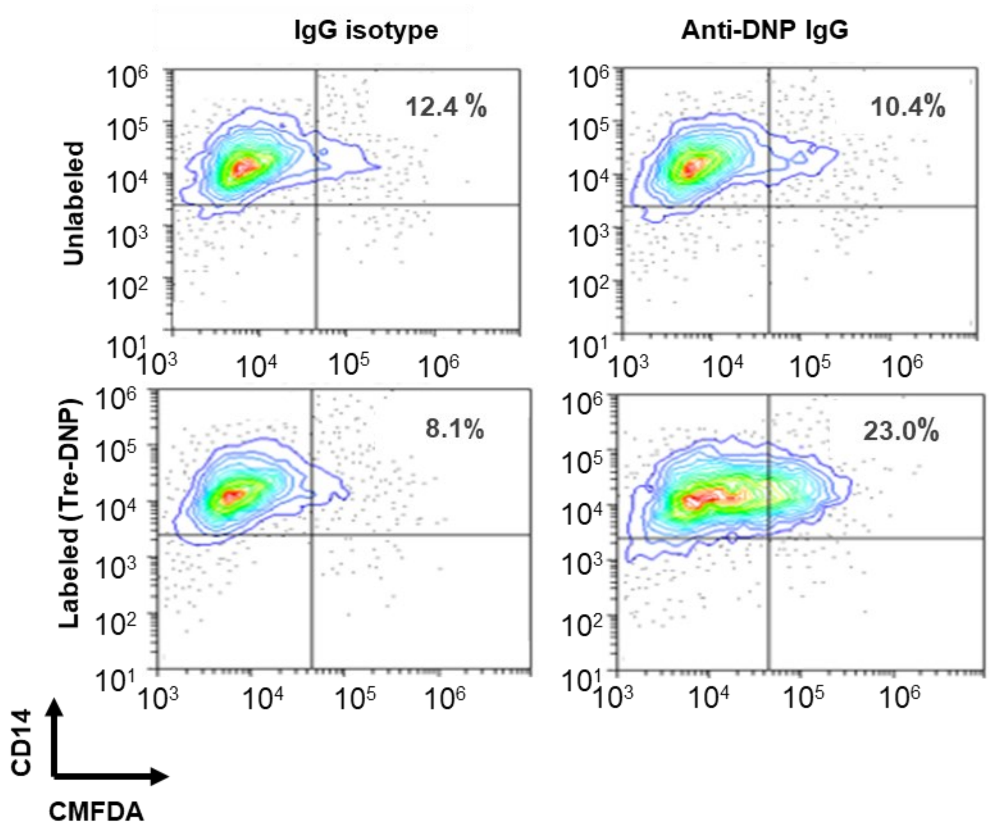

Figure S4. Representative flow cytometry contour plots and gating strategy for phagocytosis experiments. Mabs was labeled with Tre-DNP (250 μM) (or left unlabeled), washed, stained with CMFDA (10 μM), washed, and treated with mouse anti-DNP antibody (or left untreated). Bacteria (1 × 10^7 CFU/mL) were co-cultured with PMA-differentiated THP-1 cells (1 × 10^6 cells/mL). After staining macrophages with anti-CD14-APC, phagocytosis of CMFDA-labeled bacteria was analyzed by flow cytometry.

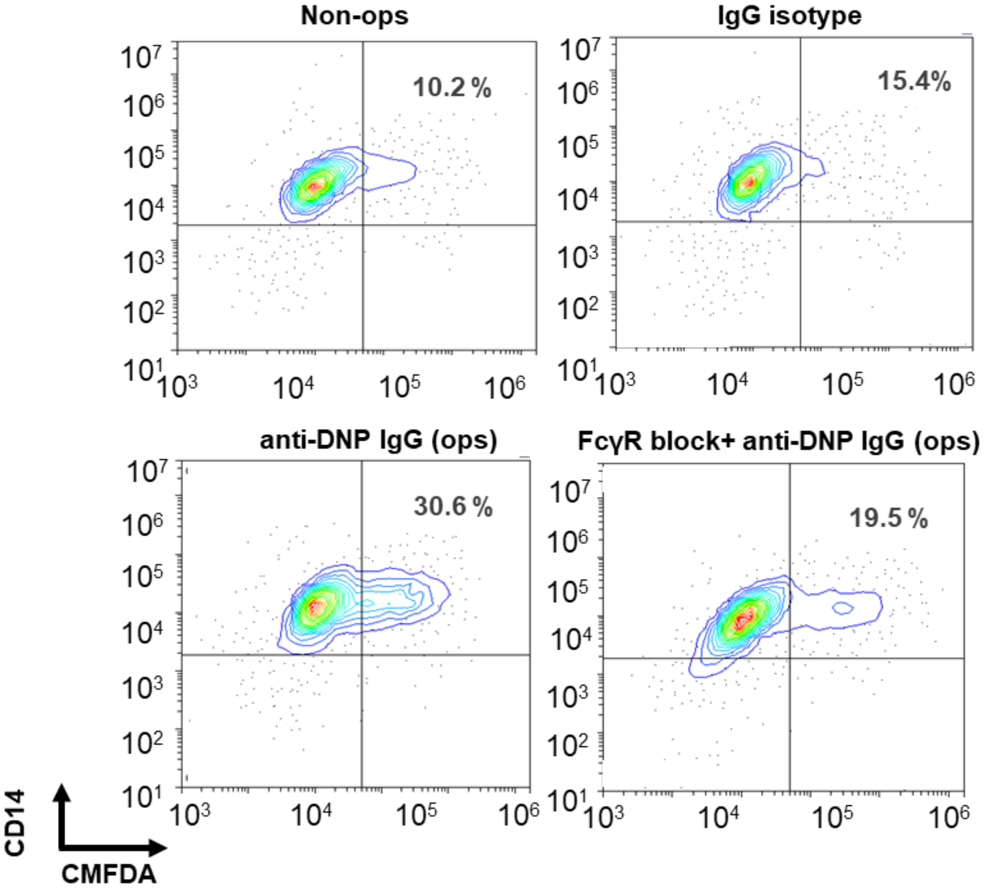

Figure S5. Representative flow cytometry contour plots and gating strategy for FcγR-dependent phagocytosis experiments. Mabs was labeled with Tre-DNP (250 μM) (or left unlabeled), washed, stained with CMFDA (10 μM), washed, and treated with mouse anti-DNP antibody (or isotype control antibody or left untreated). Bacteria (1×10^7 CFU/mL) were co-cultured with PMA-differentiated THP-1 cells (1×10^6 cells/mL). For FcγR blocking condition, PMA-differentiated THP-1 cells were pre-treated with FcγR blocker for 30 min prior to co-culturing with bacteria. After staining differentiated THP-1 cells with anti-CD14-APC, phagocytosis was analyzed by flow cytometry.

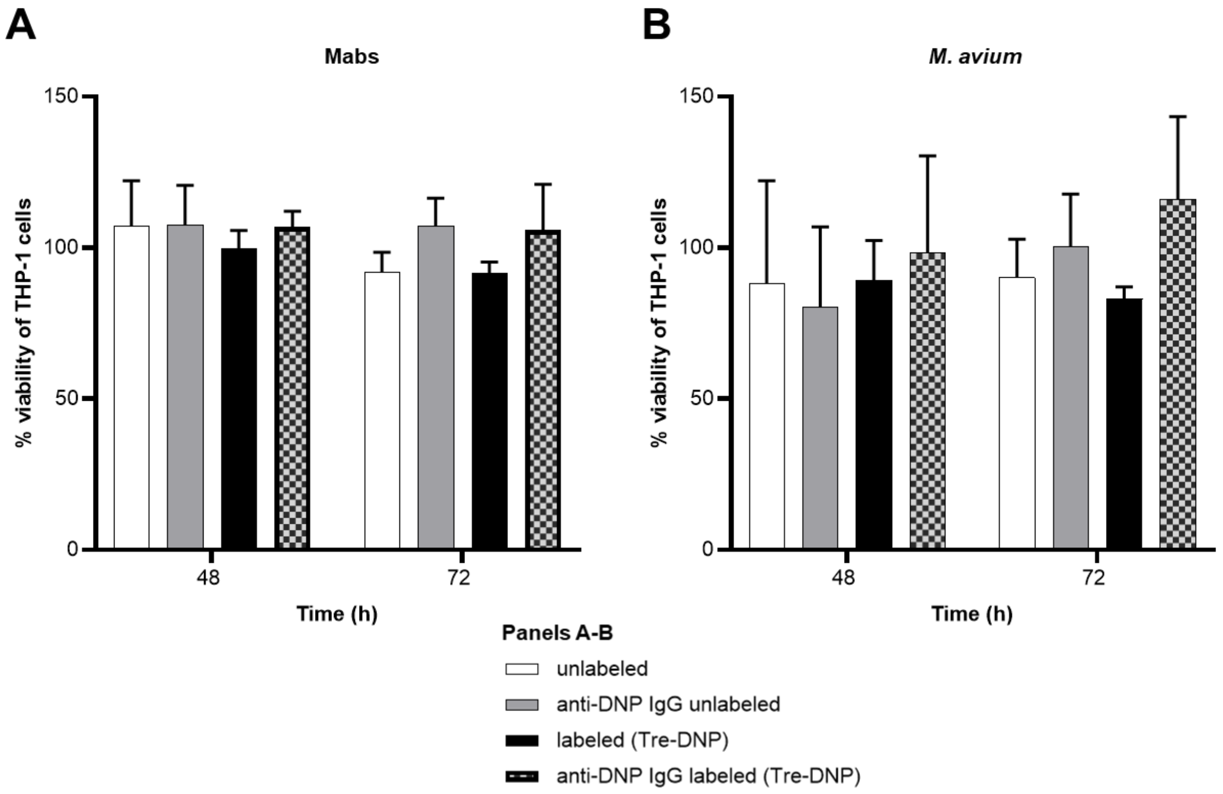

Figure S6. Intracellular killing of mycobacteria was not due to THP-1 cell death. Mabs and *M. avium*, either Tre-DNP-labeled or left unlabeled, were treated with or without mouse anti-DNP antibody. The bacteria (2×10^6 CFU/mL) were co-cultured with PMA-differentiated THP-1 cells (2×10^5 cells/mL) for 1 h. Extracellular bacteria were killed by treatment with amikacin (200 µg/mL). THP-1 cell viability was assessed at 48 h and 72 h by treating with Alamar Blue (10%) for 4 h, followed by fluorescence readings taken with a microplate reader. The percentage of mycobacteria-infected THP-1 cell viability was determined relative to uninfected THP-1 cells. (A-B) Viability of Mabs- and *M. avium*-infected THP-1 cells, respectively. Statistical analysis was performed using a two-way ANOVA with Turkey’s multiple comparison test.

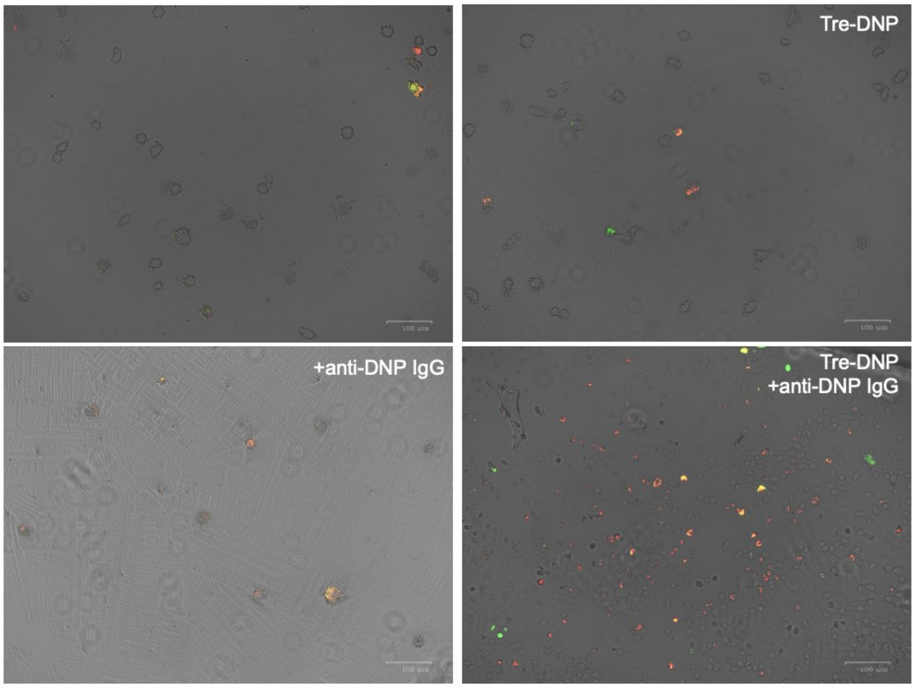

Figure S7. Representative images for phagolysosome fusion experiments. Mabs was either labeled with Tre-DNP or left unlabeled and stained with 20 μM CMFDA (green). Bacteria were then incubated in buffered saline or opsonized with anti-DNP antibody. The bacteria (2 × 10^6 CFU/mL) were co-cultured with J774 murine macrophages (4 × 10^5 cells/mL) for 1 h, and extracellular bacteria were eliminated with 100 μg/mL gentamicin. At each time point, cells were stained with 0.5 μM LysoTracker Deep Red (red) and imaged by fluorescence microscopy. Shown are representative overlay images of macrophages 1 h post infection (scale bar = 100 μM).
